# Huayu Wan enhances immune checkpoint inhibitor efficacy in triple-negative breast cancer by normalizing tumor vasculature and remodeling the tumor immune microenvironment

**DOI:** 10.3389/fimmu.2026.1824721

**Published:** 2026-05-19

**Authors:** Wenjing Yang, Jiafeng You, Hongkai Zhang, Junfan Wu, Xinran Guo, Dingkun Guan, Guowang Yang

**Affiliations:** 1 Beijing Hospital of Traditional Chinese Medicine, Capital Medical University, Beijing, China; 2 School of Clinical Medicine, Beijing University of Chinese Medicine, Beijing, China; 3 Yanqing Hospital of Beijing Hospital of Traditional Chinese Medicine, Capital Medical University, Beijing, China

**Keywords:** Huayu Wan (HYW), hypoxia, immune checkpoint inhibitors (ICIs), triple-negative breast cancer (TNBC), tumor immune microenvironment (TIME), tumor vascular normalization

## Abstract

**Background:**

Huayu Wan (HYW), a Traditional Chinese Medicine (TCM) formulation widely used in the clinical treatment of cancer, has demonstrated anti-tumor activity and augments chemotherapy efficacy by improving intratumoral drug delivery in non-small cell lung cancer. However, its efficacy and role in enhancing the response to immune checkpoint inhibitors (ICIs) for the treatment of triple-negative breast cancer (TNBC) remain unclear.

**Objective:**

To study the underlying mechanisms of HYW in enhancing the efficacy of ICIs for TNBC treatment.

**Materials and methods:**

In the 4T1 TNBC model, tumor growth, vascular structure/function, immune cell infiltration, angiogenic factors and effector cytokines were evaluated using ultrasound imaging, immunohistochemistry, immunofluorescence, and ELISA. HYW’s effects on angiogenesis were validated via *in vitro* assays (HUVEC proliferation, tube formation, wound healing and transwell invasion) under normal oxygen/hypoxic conditions.

**Results:**

TCGA data and network pharmacology identified VEGFA and the PI3K-Akt/HIF-1 signaling axis as key targets of HYW. HYW significantly enhanced ICIs efficacy, with a tumor inhibition rate of 36.55% (vs. 5.01% for ICIs alone). Mechanistically, HYW: Normalized tumor vasculature: Restored the balance between proangiogenic and antiangiogenic factors (reduced VEGFA/bFGF, increased TSP-1/PF4), enhanced pericyte coverage and improved vascular maturity, and enhanced intratumoral perfusion (ultrasound imaging). Alleviated hypoxia: Inhibited HIF-1α and reduced hypoxic regions (Hypoxyprobe staining). Remodeled the TME: Promoted CD8^+^ T cell infiltration, increased effector cytokines (GZMB, IFN-γ, TNF-α), and inhibited CD8^+^ T cell exhaustion, downregulated exhaustion markers (Tim3, CD244, CD101, PD-L1). Inhibited PI3K-Akt-mTOR signaling: Reduced downstream HIF-1α/VEGFA expression, breaking the hypoxia-angiogenesis-immunosuppression cycle.

**Conclusion:**

HYW enhances ICIs efficacy in TNBC by normalizing tumor vasculature and remodeling the tumor immune microenvironment (TIME), which provides a novel rationale for combining TCM with ICIs to improve TNBC treatment outcomes.

## 1 Introduction

TNBC, a highly aggressive subtype accounting for approximately 15-20% of all breast cancers (1, 2), is defined by the absence of estrogen receptor (ER), progesterone receptor (PR), and human epidermal growth factor receptor 2 (HER2) expression. These molecular features render TNBC non-responsive to conventional hormonal and targeted therapies, leading to higher recurrence rates and poorer prognosis compared to other breast cancer subtypes (3, 4). In recent years, ICIs have achieved remarkable efficacy in multiple refractory solid tumors by leveraging the immune system to identify and eradicate tumor cells. Owing to its higher tumor infiltrating lymphocytes (TILs) (5–9), higher PD-L1 expression (10, 11), and higher tumor mutational burden (TMB) (12, 13), TNBC has emerged as a preferred subtype for ICIs (14). Although Food and Drug Administration (FDA) and Chinese Society of Clinical Oncology (CSCO) guidelines have approved immunotherapy-based treatment for both advanced and early-stage (II–III) TNBC (15–20), most patients exhibit poor responses to ICIs alone, with an overall response rate (ORR) of less than 20% (21). This highlights an urgent need to explore novel therapeutic strategies to enhance the efficacy of ICIs in TNBC.

It is well established that tumors cannot grow beyond 2–3 mm in diameter without neovascularization, highlighting the critical role of blood vessels in tumor progression (21–24). Furthermore, tumor blood vessels are a key component of the TME (25). They serve as conduits for nutrient and oxygen supply while facilitating the delivery of anticancer agents (chemotherapy, targeted therapy, ICIs, and so on) and immune cells (CD8^+^ T cells, NK cells, macrophages, and so on) into the tumor parenchyma (26)-processes that are essential for ICIs responsiveness. Under physiological conditions, the balance between proangiogenic factors and antiangiogenic factors maintains vascular homeostasis. In the tumor microenvironment, however, tumor and stromal cells secrete multiple proangiogenic factors (VEGFA as the primary driver) (27, 28), disrupting this balance and leading to structural abnormalities (abnormally shaped, dilated with sacs, tortuous, chaotic and likely to have dead ends) and functional impairments (disturbed hemodynamics and increased vascular permeability) (27, 28). These vascular defects not only reduce the delivery of anticancer drugs and immune cells into tumors, but also promote a hypoxic TME, which further upregulates VEGFA secretion, forming a vicious cycle. Collectively, these vascular-related perturbations contribute to the establishment of a locally immunosuppressive microenvironment. Notably, vascular structural/functional abnormalities and hypoxia are more pronounced in TNBC than in other breast cancer subtypes (29), significantly limiting the efficacy of ICIs in this population. “Tumor vascular normalization”, which aims to normalize aberrant tumor vasculature, restore stable blood flow and perfusion, and thereby enhance the intratumoral delivery of ICIs and immune cells, improving the immunosuppressive TME into an immunesupportive TME. This offers a promising solution to overcome the aforementioned clinical obstacles in TNBC immunotherapy. Preclinical and clinical studies have confirmed that vascular normalization can significantly improve ICIs-mediated antitumor efficacy (30–35).

HYW is a TCM formulation developed by Professor Yu Rencun at the Beijing Hospital of Traditional Chinese Medicine, Capital Medical University. Comprising 15 medicinal herbs: *Crassostrea gigas*, *Hirudo nipponia*, *Tabanus*, *Amomum villosum Lour.*, *Persicae semen*, *Paeonia lactiflora Pall.*, *Astragalus membranaceus* Fisch. ex Bunge, *Angelica dahurica* (Hoffm.) Benth. & Hook.f. ex Franch. & Sav., *Carthamus tinctorius L.*, *Curcuma zedoaria* (Christm.) Roscoe, *Curcuma wenyujin* Y.H.Chen & C.Ling, *Corydalis yanhusuo* (Y.H.Chou & Chun C.Hsu) W.T.Wang ex Z.Y.Su & C.Y.Wu, *Panax ginseng* C.A.Mey., *Bistorta officinalis Delarbre*, *Vaccaria segetalis* (Neck.) Garcke ex Asch. It is formulated to exert the therapeutic effects of “Yiqi Huoxue Jiedu” (YQHXJD; replenishing qi, activating blood circulation, and detoxifying). Clinically, HYW is widely used in the treatment of breast cancer, lung cancer, and ovarian cancer, with proven efficacy in inhibiting tumor progression and improving patient quality of life (36). *In vivo* studies (37–41) have demonstrated that HYW significantly suppresses metastasis in 4T1 TNBC and Lewis lung cancer models. Mechanistically, HYW enhances chemotherapy efficacy by increasing intratumoral drug delivery, consistent with the “tumor vascular normalization” theory. However, the therapeutic potential of HYW in combination with ICIs for TNBC and its underlying mechanisms remain unclear. Thus, this study aimed to investigate the synergistic efficacy and molecular mechanisms of HYW combined with ICIs in TNBC.

## 2 Materials and methods

### 2.1 TCGA database analysis

The RNA sequencing (RNA-Seq) transcriptome data and corresponding clinical information of breast cancer were downloaded from The Cancer Genome Atlas (TCGA) database (https://cancergenome.nih.gov/). Patients with TNBC (ER-, PR- and HER2-) were selected for further analysis. Differential expression analysis between TNBC and normal groups was performed using the limma R package, and DEGs were defined with the cutoff criteria: fold change log2|FC| ≥ 1 and FDR < 0.05. To evaluate VEGFA expression differences between TNBC and normal tissues, normalized expression values of VEGFA were compared. TNBC patients were stratified into high- and low-VEGFA expression groups based on the median VEGFA expression level, then GSEA was conducted to investigate the association between VEGFA expression and the “ANGIOGENESIS” gene set, and the number of permutations was set to 1,000 and a false discovery rate (FDR) <0.25 was recognized as statistically significant.

### 2.2 Materials

Hypoxyprobe™ RedAPC Kit (Cat# HP8-x) was purchased from HPI (Burlington, USA), anti-mouse PD-1 was purchased from Bio X Cell (RMP1-14) (Burlington, USA), Sulfo-Cyanine5.5 was purchased from MedChem Express (New Jersey, USA), Matrigel (356234) was purchased from BD (New Jersey, USA). PerCP/Cyanine5.5 anti-mouse CD45, Alexa Fluor^®^ 700 anti-mouse CD3, FITC anti-mouse CD4, PE/Cyanine7 anti-mouse CD8a, PE anti-mouse CD137, APC anti-mouse CD279 and Zombie NIR™ Fixable Viability Kit were all purchased from Biolegend (California, USA). Anti-mouse CD31/a-SMA/VE-cadherin/CD8 were all purchased from Servicebio (Wuhan, China). Anti-mouse PD-L1 was purchased from Proteintech (Wuhan, China). Secondary antibodies included goat anti-mouse Alexa Fluor^®^ 488 and Cy3, HRP-labeled goat anti-rabbit were all purchased from Servicebio (Wuhan, China). Anti-mouse PI3K/AKT/p-AKT/mTOR/p-mTOR/hypoxia inducible factor-1a (HIF-1α) were all purchased from Cell Signaling Technology (Boston, USA). anti-mouse VEGFA was purchased from Abcam (Cambridge, UK), anti-mouse β-tubulin was purchased from Servicebio (Wuhan, China). Secondary antibodies included HRP Conjugated AffiniPure Goat Anti-rabbit IgG (H+L), mouse VEGFA/granzyme B (GZMB)/interferon-gamma (IFN-γ)/tumor necrosis factor-alpha (TNF-a) ELISA Kit were all purchased from Boster Biological Technology (Wuhan, China), mouse basic fibroblast growth factor (bFGF)/platelet factor 4 (PF4)/thrombospondin-1 (TSP-1) were all purchased from Elabscience (Wuhan, China).

### 2.3 HYW preparation

The HYW formula granules were provided by Beijing Hospital of Traditional Chinese Medicine, Capital Medical University (Beijing, China), consisting of 15 common Chinese herbal extracts as listed in **Table 1**. To ensure the quality consistency of HYW extract, all HYW used in our experiments was purchased from the same batch of raw materials and extracted under identical conditions following a standardized process. The botanical names were validated using the website (http://www.worldfloraonline.org and http://mpns.kew.org), with the verification date of January 25, 2026.

**Table 1:** The composition of HYW.

English name	Chinese name	Lot number
*Amomum villosum Lour.*	Sharen	23092804
*Persicae semen*	Taoren	24031201
*Paeonia lactiflora Pall.*	Chishao	24070501
*Astragalus membranaceus* Fisch. ex Bunge	Huangqi	24092001
*Angelica dahurica* (Hoffm.) Benth. & Hook.f. ex Franch. & Sav.	Baizhi	24090901
*Carthamus tinctorius L.*	Honghua	24080801
*Curcuma zedoaria* (Christm.) Roscoe	Ezhu	2406119
*Curcuma wenyujin* Y.H.Chen & C.Ling	Yujin	2407124
*Corydalis yanhusuo* (Y.H.Chou & Chun C.Hsu) W.T.Wang ex Z.Y.Su & C.Y.Wu	Yanhusuo	24061807
*Panax ginseng* C.A.Mey.	Hongshen	2401115
*Bistorta officinalis Delarbre*	Quanshen	240803004
*Vaccaria segetalis* (Neck.) Garcke ex Asch.	Wangbuliuxing	2409017
*Crassostrea gigas*	Muli	24052504
*Hirudo nipponia*	shuizhi	23022802
*Tabanus*	Mengchong	24092205

Previous systematic dose-gradient studies on HYW in TNBC models identified 27.3 g/kg as the optimal dose that balances efficacy and safety (39, 41), with no significant difference observed at higher doses. Furthermore, this dose has been demonstrated to effectively modulate angiogenic factors (38, 42), enhance tumor blood supply, and improve drug delivery (37) (**Supplementary File**). Therefore, to align with clinical practice and avoid redundant dose exploration, the present study employed this established dose as the core concentration for investigating vascular normalization and the underlying mechanisms of combined immunotherapy.

These herbs were mixed and extracted twice by refluxing with 10 times of water (volume/weight) for 2.0 h each time, then the extracted solution was filtered and the procedure was repeated one more time. The filtered extracts were mixed together and then concentrated to a relative density for 2.73 g/ml. In our study, HYW was administered at a normal dose corresponding to its clinical dose ( $D_{m}=\frac{D_{h}}{W}\times F$).

### 2.4 HYW drug containing serum preparation

Rats were randomly divided into two groups: HYW-treated group and normal saline (NS) control group (n = 6 rats per group). The HYW-treated group received intragastric administration of HYW at a dosage of 18.9 g/kg body weight, twice daily for 7 consecutive days. The control group was administered an equal volume of NS via the same route and schedule. The blood samples were collected from the abdominal aorta into sterile centrifuge tubes. The blood was allowed to clot at room temperature for 30 min, followed by centrifugation at 4000 r/min for 15 min. The separated serum was heat-inactivated in a water bath at 56°C for 30 min to eliminate complement activity, then filtered through a 0.22 μm sterile filter under aseptic conditions. The HYW-containing serum and control serum were aliquoted and stored at -80°C until use.

### 2.5 LC-MS analysis of HYW

The chemical constituents of HYW were separated and analyzed using liquid chromatography-mass spectrometry (LC-MS) in three sample types: HYW aqueous extract, HYW-containing pharmacological serum, and control serum. Chromatographic Conditions: Column: Waters HSS T3 column (100 × 2.1 mm, 1.8 μm); Column temperature: 40°C; Mobile phase: Solvent A: Milli-Q water with 0.1% formic acid, Solvent B: Acetonitrile with 0.1% formic acid; Flow rate: 0.3 mL/min; Injection volume: 2 μL; Elution gradient: 0–1 min: Isocratic at 100% A; 1–12 min: Linear gradient to 5% A/95% B; 12–13 min: Hold at 5% A/95% B; 13.1 min: Immediate return to 100% A; 13.1–17 min: Hold at 100% A.

High-resolution mass spectrometric conditions: Ion source: Heated electrospray ionization (HESI); Acquisition mode: Full-scan MS/ddMS² (data-dependent MS²); HESI source parameters: Sheath gas flow rate: 40 arbitrary units (arb), Auxiliary gas flow rate: 10 arb, Spray voltage: +3.0 kV (positive ion mode); -2.8 kV (negative ion mode), Capillary temperature: 350°C, Ion transfer tube temperature: 320°C; MS scan parameters: Full-scan range: m/z 70-1050, Full-scan resolution: 70,000 (at m/z 200), ddMS² resolution: 17,500 (at m/z 200).

### 2.6 Network pharmacology-based analysis of HYW

The main molecular structures of HYW were input into the Swiss Target Prediction database (https://www.swisstargetprediction.ch) (43) to predict their potential protein targets, and targets with a credibility score of 0 were excluded to ensure reliability. The Venny 2.1.0 (https://bioinfogp.cnb.csic.es/tools/venny/) (44) was used to visualize the overlapping targets between HYW and TNBC. The STRING database (https://cn.string-db.org/) (45) was used to construct a protein-protein interaction (PPI) network with a confidence score threshold >0.7. Network visualization was performed using Cytoscape software (version 3.10.0; https://cytoscape.org/). The Database for Annotation, Visualization and Integrated Discovery (DAVID, version 6.8; https://davidbioinformatics.nih.gov/) (46, 47) was used to perform Gene Ontology (GO) functional annotation (including biological process, cellular component, and molecular function) and Kyoto Encyclopedia of Genes and Genomes (KEGG) pathway enrichment analyses. The results were visualized using the Sanger Box online platform (http://www.sangerbox.com/) (48).

### 2.7 Cell lines and animal model

The murine breast cancer 4T1 cell line was obtained from the Shanghai Institute of Cell Biology, Chinese Academy of Sciences. Human umbilical vascular endothelial cells (HUVECs) were purchased from the China Infrastructure of Cell Line Resource. All cells were cultured in DMEM (Gibco) supplemented with 10% FBS (Gibco). 4T1 cells were cultured in a humidified atmosphere (5% CO_2_, 21% O_2_, 37°C). HUVECs were cultured in a humidified atmosphere (5% CO_2_, 21% O_2_, 37°C) and a humidified atmosphere (5% CO_2_, 1% O_2_, 37°C).

**Figure 1:**
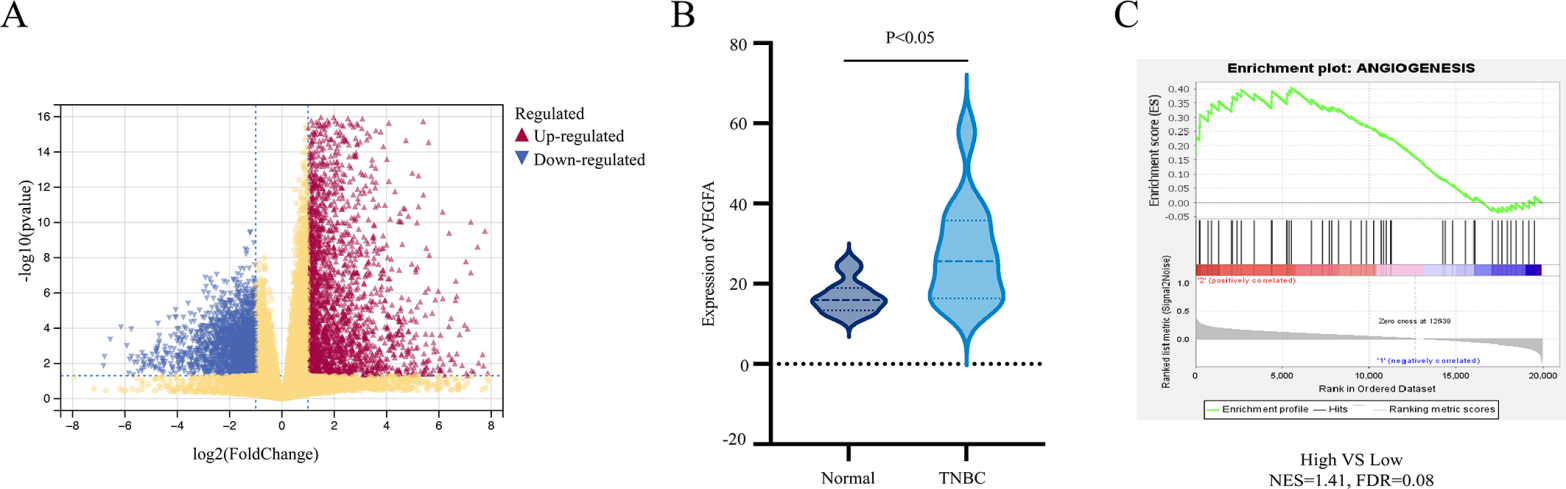
Bioinformatics analysis of angiogenesis-related genes in TNBC. **(A)** Volcano plot of the DEGs between TNBC and normal; **(B)** The mRNA expression of VEGFA between TNBC and normal; **(C)** The analysis of “ANGIOGENESIS” pathways between TNBC and normal by GSEA.

BALB/c mice (6–8 weeks, 18–20 g, female) were purchased from Beijing Vital River Laboratory Animal Technology Co., Ltd. All animal experiments were approved by the Animal Ethics Committee of Beijing Institute of Traditional Chinese Medicine (Approval No.: BJTCM-M-2024-04-06) and conducted in accordance with the guide for the care and use of laboratory animals. All mice were carefully bred in specific pathogen-free barrier facilities at the Beijing Institute of Traditional Chinese Medicine, and the 4T1 TNBC model was established as previously reported (49), then the animals were randomly divided into model group, anti-PD-1 group, HYW group, anti-PD-1 + HYW group, a normal group (no intervention) was set up at the same time, each group included 5 mice. HYW group and anti-PD-1 + HYW group were administered HYW aqueous extract (2.73 g/mL, 0.2mL) by oral gavage once a day. anti-PD-1 group and anti-PD-1 + HYW group was administered anti-PD-1 (7.5 mg/kg) by intraperitoneal injection every three days (at the 11th day, 14th day, 17th day, 20th day and 23rd day) (**Figure 1A**).

### 2.8 Immunohistochemistry

The breast tumor tissues were fixed by 4% paraformaldehyde (PFA), at 4°C for 24 h, followed by dehydration through a graded ethanol series, paraffin embedding, and sectioning into 4-μm-thick slices, then stained with H&E (10 min hematoxylin and 1 min eosin) or incubated with anti-PD-L1, three randomly selected microscopic fields per sample, and positive signals were further evaluated using ImageJ software.

### 2.9 Preparation of CY5.5-anti-PD-1

A total of 12 µL CY5.5 stock solution (10 mg/mL, 0.1 mL) was mixed with 60 µL activation buffer, then added to 1 mL anti-PD-1 solution (2 mg/mL, 1 mL) and gently vortexed. Incubated on a shaking platform at 4°C for 24 hours in the dark. The mixture was aliquoted into four ultrafiltration units (0.5 mL, 30 kDa molecular weight cutoff, PES membrane), 13,000 g, 10 minutes, quantified by the BCA Protein Assay Kit, and stored at 4°C.

### 2.10 Immunofluorescence

The breast tumor tissues were fixed with 4% PFA and sectioned, and incubated with anti-mouse CD31 (1:500)/α-SMA (1:300)/VE-cadherin/CD8^+^ T (1:3000) overnight, and then incubated with CY3-labeled secondary antibody (1:300)/Alexa Fluor 488-conjugated secondary antibody (1:400)/HRP -conjugated secondary antibody (1:500) for 50 min at 37°C, respectively. Nuclear counterstaining was performed with DAPI. To evaluate the delivery efficacy of HYW on anti-PD-1, mice received intraperitoneal injection of CY5.5-anti-PD-1 (7.5 mg/kg) 6 hours before euthanasia, under constant inhalation with isoflurane at 2-3%, followed by blood collection via cardiac puncture. Tissues were incubated with anti-mouse CD31 (1:500) overnight, and then incubated with CY3 (1:300) conjugated secondary antibodies. Three randomly selected microscopic fields per sample, and positive signals were further evaluated using ImageJ software.

### 2.11 Transmission electron microscope imaging

Breast tumor tissues were fixed with 2.5% electron microscopy fixative overnight, then perform three rinses with 0.1 M Phosphate Buffer (PB), re-fix with 1% osmium tetroxide solution, graded dehydration, and embedded in epoxy resin. Then sections (80 nm) were stained with 2.6% lead citrate solution. And digital images were acquired using transmission electron microscope.

### 2.12 Cell proliferation, tube formation assay, wound healing and transwell invasion

Cell proliferation: The HUVECs viability was evaluated using CCK-8 assays. HUVECs were seeded at 2,000 cells/well in 96-well plates, and incubated overnight under normal oxygen conditions (5% CO_2_, 21% O_2_) and hypoxic conditions (5% CO_2_, 1% O_2_). After drug treatment (HYW drug containing serum or control serum at concentrations of 10%),100μl of CCK-8 working solution (prepared at a ratio of 9:1 with complete culture medium) was transferred into the culture plates at 24 hours, 48 hours, and 72 hours respectively, then incubated for 1.5 hours at 37°C in the dark. And optical density (OD) value was measured at absorbance wavelength of 450 nm.

Tube formation assay: 48-well plates were coated with Matrigel (150 µl/well) and incubated at 37°C for 1 hour, then HUVECs were seeded at 80,000 cells/well in a low serum medium supplemented with HYW drug containing serum or control serum at concentrations of 10%, then incubated under normal oxygen conditions (5% CO_2_, 21% O_2_) and hypoxic conditions (5% CO_2_, 1% O_2_), with three replicate wells, and incubated at 37°C for 4 hours. The images were captured with inverted microscope, the number of branch points within the tubular networks was quantified in three randomly selected fields of view using ImageJ software.

Wound Healing: HUVECs were seeded at 1×10^6^ cells/well in 6-well plates, and incubated overnight under normal oxygen conditions (5% CO_2_, 21% O_2_) and hypoxic conditions (5% CO_2_, 1% O_2_). After reaching a confluence of 100%, the cell monolayers were scratched using a 200-µl pipette tip and then the floating cells were removed with PBS. After drug treatment (HYW drug containing serum or control serum at concentrations of 10%), images were captured with inverted microscope at 0 hours and 8 hours, and the wound healing rate was analyzed using ImageJ software.

Transwell invasion: HUVECs were seeded into upper inserts at 10,000 cells/well in a low serum medium supplemented with HYW drug containing serum or control serum at concentrations of 10%, and adding complete culture medium with 4T1 cells to the lower chamber, then incubated under normal oxygen conditions (5% CO_2_, 21% O_2_) and hypoxic (5% CO_2_, 1% O_2_) conditions, with three replicate wells, and incubated at 37°C for 24 hours. HUVECs were removed on the upper surface of the transwell membrane with cotton swabs, were fixed with 4% paraformaldehyde for 15 min, were stained with crystal violet dye solution for 30 min, were washed with PBS, were mounted on microscope slides. And images were captured with inverted microscope and quantified the number of HUVECs in three randomly selected fields of view using ImageJ software.

### 2.13 Animal ultrasound imaging system

The animal ultrasound imaging system (S-Sharp Corporation) was used to measure the directional blood flow rate (Doppler mode), depicting the two-dimensional blood vessel distribution (Doppler mode) and quantitatively analyzing the local blood flow velocity (PW-mode), which may detect the blood perfusion dynamics in tumor tissue from multiple dimensions and was performed as previously reported (at the 11th day, 14th day, 17th day, 20th day and 23rd day) (50). The raw data were extracted for subsequent analysis.

### 2.14 The hypoxic detection

The Hypoxyprobe™ Red APC Kit (cat. no. HP8-x) contains pimonidazole hydrochloride and Hypoxyprobe-1 Mab1 mouse monoclonal antibody, which was used in the present study to detect hypoxia in breast tumors as previously reported.

### 2.15 Transcriptomics by RNA-sequencing

Total RNA was isolated from breast tumor tissues using TRIzol^®^ Reagent (Invitrogen), RNA concentration and purity were quantified using NanoDrop™. Second-generation high-throughput sequencing was conducted using the DNBSEQ-T7 system (BGI Genomics, Shenzhen, China): Total RNA was fragmented into short fragments, and mRNA was enriched using oligo (dT) magnetic beads. This was followed by cDNA synthesis, and the double-stranded cDNA was purified and enriched through PCR amplification. The library products were then sequenced using BGIseq-500. Differentially expressed genes (DEGs) were identified using the limma R package (v3.54.0) with the following thresholds: fold change log|FC| ≥ 1 and FDR < 0.05. DAVID database (https://david.ncifcrf.gov) was used to perform KEGG pathway enrichment analyses, and the results were visualized by Sanger Box (http://www.sangerbox.com/). GSEA was performed in “ANGIOGENESIS”/“HIF” pathways between HYW group and Model group, and the number of permutations was set up to 1,000 and a false discovery rate (FDR) <0.25 was recognized as statistically significant.

### 2.16 Deconvolution using TIMER

TIMER was used to analyze RNA sequencing expression profiles normalized by quantile normalization (transcripts per million, TPM). Subsequently, the relative infiltration ratio of CD8^+^ T cells in each group was quantified using the Immune Oncology Biomarker Research (IOBR) R package.

### 2.17 ELISA assay

The plasma concentrations of VEGFA/bFGF/TSP-1/PF4/GZMB/IFN-γ/TNF-a were quantified using commercially available anti−mouse ELISA kits, adhering strictly to the manufacturer’s guidelines. All assays were run in duplicate at a suitable dilution. Absorbance readings were taken at wavelengths of 450 nm.

### 2.18 Western blot analysis

Breast tumor tissues were lysed in ice-cold RIPA buffer containing 1% phosphatase and 1% protease inhibitor for 30 min, were centrifuged for 10 min (12,000 g, 4°C), were quantified by BCA protein assay kit, and were denatured at 95°C for 5 minutes. Equal amounts of proteins (30 µg/lane) were subjected to SDS-PAGE and then electronically transferred onto a PVDF membrane. Membranes were then blocked with TBS and 0.1% Tween-20 (TBST) containing 10% non-fat milk at room temperature for 1 h and incubated overnight with the following antibodies at 4°C: PI3K (1:1000 dilution), AKT (1:1000 dilution), p-AKT (1:1000 dilution), mTOR (1:1000 dilution), p-mTOR (1:1000 dilution), HIF-1α (1:1000 dilution), VEGFA (1:1000 dilution), and β-tubulin (1:1000 dilution). After washing with TBST 3 times, the membrane was incubated in HRP-conjugated secondary antibody for 1 h at room temperature, washed with TBST again for 10 min at room temperature and visualized in a dark room. The protein bands were developed by an enhanced chemiluminescence (ECL) detection reagent and recorded with the SCG-W3000 PLUS imaging system. And the grey value of each band was measured using ImageJ software.

### 2.19 Statistical analysis

Statistical evaluations data visualization was performed using GraphPad Prism 10.0 software. All quantitative data are presented as mean ± standard deviation (SD). For comparisons between two groups, unpaired two-tailed Student’s t-test was applied, while one-way analysis of variance (ANOVA) followed by Tukey’s *post-hoc* test was used for multiple group comparisons. Statistical significance was defined as a two-tailed p-value < 0.05.

## 3 Results

### 3.1 Bioinformatics analysis of angiogenesis-related genes in TNBC

To investigate angiogenesis-associated gene expression patterns in TNBC, transcriptomic data of 132 TNBC patients meeting the inclusion criteria were retrieved from TCGA. Differential expression analysis between TNBC tissues and adjacent normal breast tissues identified 2,922 DEGs, consisting of 1,371 upregulated and 1,551 downregulated genes (**Figure 1A**). Among these, VEGFA expression was significantly elevated in TNBC tissues compared to normal controls (P < 0.05; **Figure 1B**). Subsequently, the 132 TNBC patients were stratified into two cohorts based on the median VEGFA expression level: a high-expression group (n = 66) and a low-expression group (n = 66). GSEA revealed that the high-VEGFA group was significantly enriched in the “ANGIOGENESIS” gene set (NES = 1.41, FDR = 0.08; **Figure 1C**), indicating a strong correlation between VEGFA overexpression and angiogenic pathway activation in TNBC.

### 3.2 Network pharmacology-based analysis of HYW

**Figure 2:**
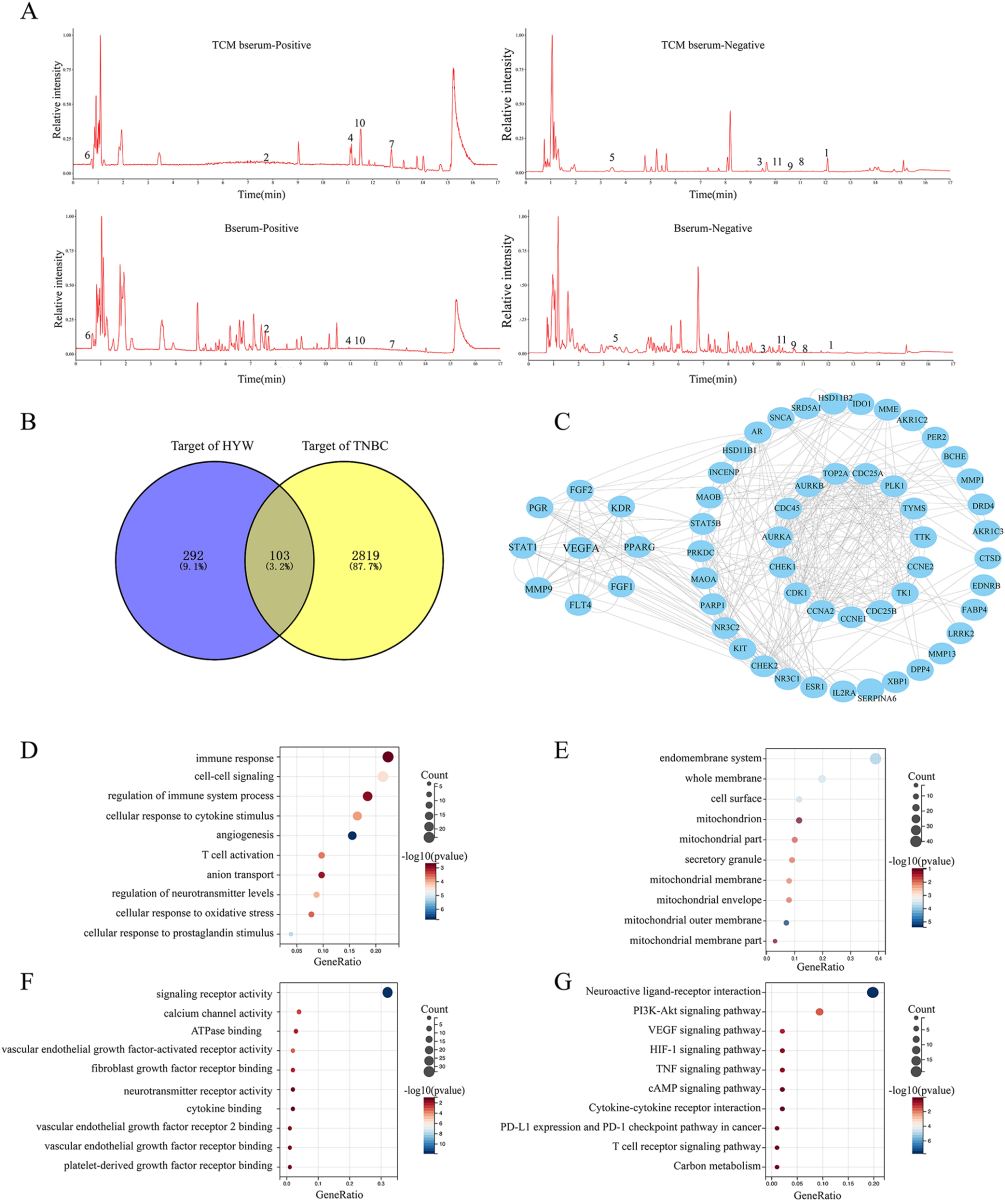
Network pharmacology-based analysis of HYW. **(A)** LC-MS analysis of HYW; **(B)** Venny diagram of HYW and TNBC targets; **(C)** PPI network of common targets of HYW and TNBC. **(D–F)** GO analysis; **(G)** KEGG pathway enrichment analysis.

To ensure effectiveness and stability of HYW quality for pharmacological evaluation, the contents of major bioactive compounds were quantified. A total of 109 chemical constituents were initially isolated based on the following criteria: fragmentation score >80 and serum concentration >2×10^5^ ng/μL, the chemical structures and quantitative results of these 11 components are presented in (**Figure 2A**) and **Table 2**. Canonical SMILES corresponding compounds were searched in the PubChem database. Potential targets of these constituents were predicted using SwissTargetPrediction. A total of 292 potential targets of HYW active ingredients were obtained. The 103 overlapping genes which represent the potential therapeutic targets between HYW targets and TNBC targets were identified by Venny 2.1.0 and shown in (**Figure 2B**). The PPI network diagram of HYW for TNBC which was visualized by Cytoscape 3.10.0 was shown in (**Figure 2C**), and the core target VEGFA were identified. The top 10 enriched GO terms of molecular functions, cellular components, and biological processes were shown in (**Figures 2D–F**), indicating HYW could influence immune response, positive regulation of immune system process, angiogenesis, T cell activation, cytokine binding, vascular endothelial growth factor-activated receptor activity, vascular endothelial growth factor-activated receptor activity, vascular endothelial growth factor binding, and so on. KEGG pathway enrichment analysis identified 59 key pathways, and the top 10 enriched terms were shown in (**Figure 2G**), including PI3K-Akt signaling pathway, T cell receptor signaling pathway, cytokine-cytokine receptor interactions, VEGF signaling pathway, central carbon metabolism in cancer, PD-L1 expression and PD-1 checkpoint pathways in cancer, cAMP signaling pathway, HIF-1 signaling pathway, Carbon metabolism, mTOR pathway. These results suggest that HYW may exert anti-angiogenic effects and enhance anti-PD-1 efficacy through VEGFA-mediated modulation of the PI3K-Akt/HIF-1 signaling axis.

**Table 2:** The 11 principal hemostatic components in Huayu Wan (HYW).

No.	RT (min)	Detected (m/z) [M-H]^-^	Identified compounds	Molecular formula	Fragmentation score
1	12.03	485.3289664	ganoderic acid tr	C_30_H_44_O_4_	90.3
2	7.72	430.2941695	glycocholic acid	C_26_H_43_NO_6_	89.7
3	9.35	451.3085491	ursodeoxycholic acid	C_24_H_40_O_4_	87.0
4	11.15	603.386633	cimigenoside	C_35_H_56_O_9_	86.3
5	3.46	655.2324488	maytansinol	C_28_H_37_C_l_N_2_O_8_	84.8
6	0.71	441.1661847	epiberberine	C_2_0H_18_NO_4_^+^	84.4
7	12.73	583.4179364	alisol a 23-acetate	C_32_H_52_O_6_	82.9
8	11.09	261.150006	1,2-dehydro-alpha-cyperone	C_15_H_20_O	82.8
9	10.31	588.2984183	methyl pyropheophorbide-a	C_34_H_36_N_4_O_3_	81.8
10	11.53	555.3875248	16-oxoalisol a	C_30_H_48_O_6_	80.5
11	10.07	471.3136735	antcin a	C_29_H_42_O_4_	80.2

### 3.3 Vascular structure normalization effects of HYW

**Figure 3:**
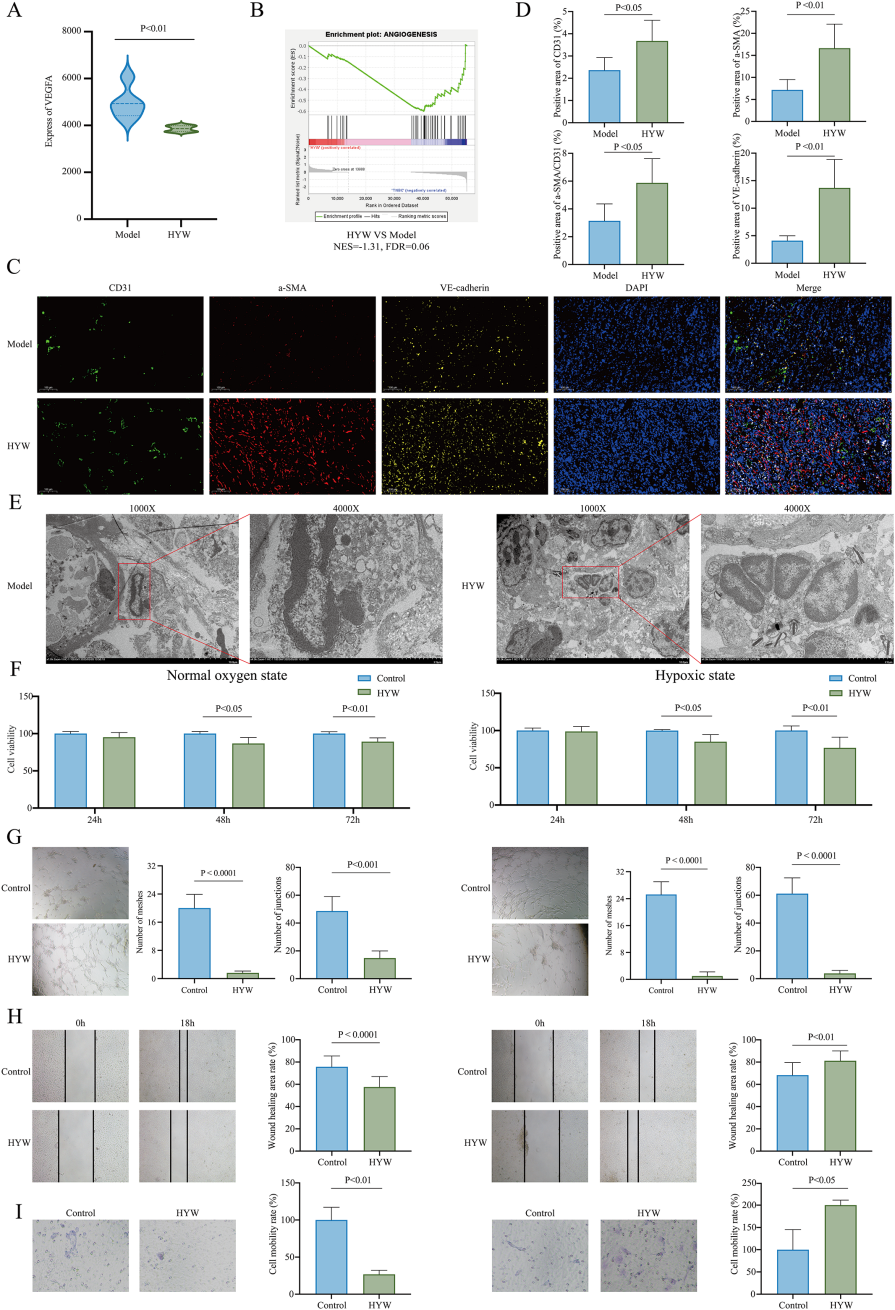
Vascular structure normalization effects of HYW. **(A)** The mRNA expression of VEGFA between HYW and normal in 4T1 TNBC; **(B)** The analysis of “ANGIOGENESIS” pathways between HYW and model by GSEA in 4T1 TNBC; **(C, D)** IF staining of CD31/α-SMA/VE-cadherin between HYW and normal; **(E)** TEM detection between HYW and normal; **(F)** Cell proliferation assay between HYW and normal under normal oxygen conditions (5% CO_2_, 21% O_2_) and hypoxic conditions (5% CO_2_, 1% O_2_); **(G)** Tube formation assay between HYW and normal under normal oxygen conditions (5% CO_2_, 21% O_2_)) and hypoxic conditions (5% CO_2_, 1% O_2_); **(H)** Wound Healing assay between HYW and normal under normal oxygen conditions (5% CO_2_, 21% O_2_)) and hypoxic conditions (5% CO_2_, 1% O_2_); **(I)** Transwell invasion assay between HYW and normal under normal oxygen conditions (5% CO_2_, 21% O_2_)) and hypoxic conditions (5% CO_2_, 1% O_2_).

To investigate whether HYW promotes tumor vascular normalization, we first detected the mRNA expression of VEGFA between model group and HYW group by transcriptome sequencing. HYW significantly reduced VEGFA mRNA expression (P < 0.01; **Figure 3A**), with significantly enriched in the “angiogenesis pathway” (NES = -1.31, FDR = 0.06; **Figure 3B**), suggesting that HYW may target VEGFA to regulate tumor angiogenesis.

Next, the vascular structure normalization was confirmed *in vivo* and *in vitro*. *In vivo*, IF staining was performed to evaluate vascular structural normalization in 4T1 tumor-bearing mice. VE-cadherin (vascular integrity marker), CD31 [endothelial cell (EC) marker], and α-SMA [pericyte (PC) marker] were co-stained to assess vascular maturity and pericyte coverage. Quantitative analysis showed that HYW significantly increased CD31^+^ EC density (P < 0.05; **Figures 3C, D**), upregulated α-SMA (P < 0.01; **Figures 3C, D**) and VE-cadherin (P < 0.01; **Figures 3C, D**) expression in the TME. Importantly, the ratio of α-SMA^+^ PCs to CD31^+^ ECs (α-SMA^+^/CD31^+^), a critical indicator of vascular stability, was significantly elevated in the HYW group (P < 0.05; **Figure 3D**). These findings indicate that HYW enhances pericyte coverage and promotes vascular maturation.

TEM was used to observe vascular ultrastructure. HYW treatment led to (**Figure 3E**): (1) increased vessel wall thickness, (2) improved morphological regularity of endothelial cells, (3) reduced abnormal vascular branching, (4) uniform lumen diameters, and (5) intact endothelial cell junctions. These ultrastructural changes collectively demonstrate that HYW restores basement membrane integrity and normalizes vascular architecture.

Integrative evidence from transcriptome sequencing, IF, and TEM confirms that HYW promotes tumor vascular structural normalization in the 4T1 TNBC model by downregulating VEGFA, enhancing pericyte coverage, improving vascular maturity, and improving vascular ultrastructural stability.

To characterize the direct impact of HYW on EC functions critical for angiogenesis, a series of *in vitro* assays were performed under normal oxygen conditions and hypoxic conditions, mimicking the dynamic oxygen tension in the TME. Cell proliferation assays (neovascularization capacity): under normal oxygen conditions (21% O_2_), HYW significantly suppressed HUVEC viability at 48 h (P < 0.05) and 72 h (P < 0.01) compared to the control group. Notably, under hypoxic conditions (1% O_2_), this inhibitory effect was further potentiated, with a more pronounced reduction in proliferation rates (**Figure 3F**). These results indicate that HYW exhibits time-dependent anti-proliferative activity against ECs, which is amplified under hypoxia. Matrigel-based tube formation assays were conducted to assess HYW’s effect on EC angiogenic sprouting. Quantitative analysis revealed that HYW treatment led to a significant decrease in the number of meshes (P < 0.0001) and junctions (P < 0.001) under normal oxygen conditions, indicating impaired formation of vascular rings and inter-ring anastomoses. Consistent with the proliferation data, hypoxic conditions exacerbated this inhibitory effect, resulting in a near-complete abrogation of tube-like structure formation (**Figure 3G**). These findings suggest that HYW directly disrupts the structural assembly of neovessels. Wound healing and Transwell assays were performed to evaluate EC migration and invasion, respectively. Under normal oxygen conditions, HYW significantly inhibited HUVEC migration (P < 0.0001; **Figure 3H**) and transwell invasion (P < 0.01; **Figure 3I**). Strikingly, under hypoxia conditions, HYW exerted the opposite effect: it significantly enhanced HUVEC migration (P < 0.01; **Figure 3H**) and invasion (P < 0.05; **Figure 3I**). This oxygen-dependent biphasic regulation suggests context-specific modulation of EC motility by HYW.

*In vitro* data demonstrate that HYW exerts dual, oxygen-dependent effects on EC functions: Under normal oxygen conditions: Inhibits proliferation, tube formation, migration, and invasion, collectively suppressing *de novo* angiogenesis. Under hypoxia conditions: Potentiates anti-angiogenic effects on proliferation and tube formation while promoting EC migration and invasion, a phenotype consistent with vascular remodeling and normalization. These findings support the hypothesis that HYW may normalize vascular structure in the hypoxic TME, thereby improving intratumoral perfusion and alleviating hypoxia, potentially contributing to optimized tumor microenvironment remodeling.

### 3.4 Vascular function normalization effects of HYW

**Figure 4:**
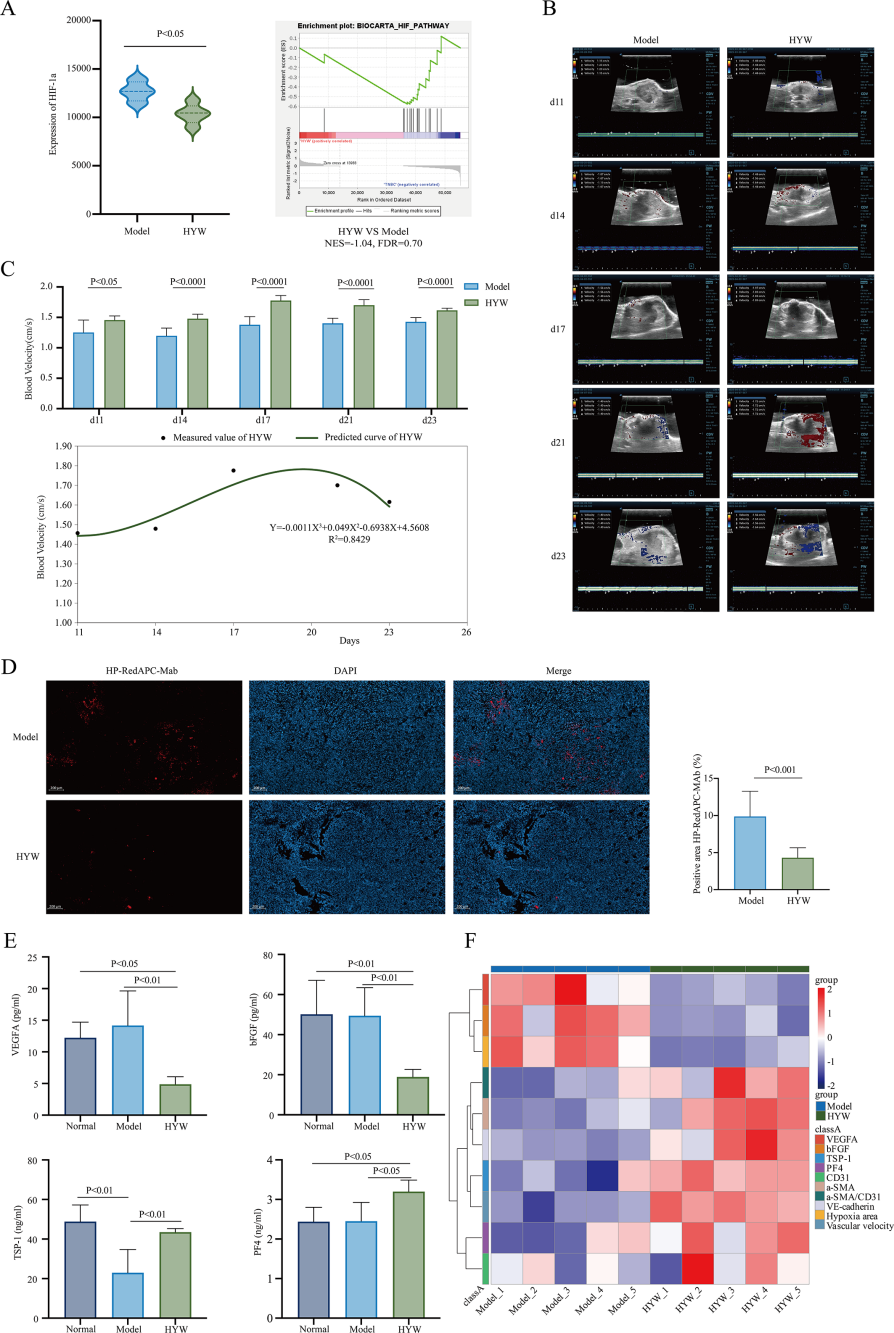
Vascular function normalization effects of HYW. **(A)** The mRNA expression of HIF-1α between HYW and model; The analysis of “HIF” pathways between HYW and normal by GSEA; **(B)** Animal ultrasound imaging system detection between HYW and normal; **(C)** Temporal analysis of flow velocity between HYW and normal; **(D)** The hypoxic detection by HP8 IHC between HYW and normal; **(E)** ELISA assay of the proangiogenic and antiangiogenic factors between HYW and normal; **(F)** Heatmap of clusters.

To investigate whether HYW modulates vascular function normalization, we systematically evaluated its impact on hypoxia signaling, tumor perfusion dynamics, and hypoxic regions in the 4T1 breast cancer model. Firstly, transcriptome sequencing was performed to profile hypoxia-related gene expression. Compared to the model group, HYW significantly reduced the mRNA expression of HIF-1α (P < 0.05; **Figure 4A**). GSEA pathway enrichment analysis further revealed enrichment in the “HIF signaling pathway” (NES = -1.04, FDR = 0.70; **Figure 4A**), suggesting that HYW may modulate the tumor hypoxic microenvironment by targeting HIF-1α target and Hypoxia related pathways.

Next, animal ultrasound imaging was used to assess vascular perfusion dynamics. HYW significantly enhanced and stabilized perfusion signals in 4T1 tumors compared to the model group (**Figure 4B**). Temporal analysis of flow velocity via polynomial curve fitting revealed a biphasic pattern in the HYW group, characterized by: (1) an initial decline, (2) subsequent augmentation, and (3) eventual reduction. The fitted cubic regression model for flow velocity was *Y* = -0.0011*X*^3^+0.049*X*^2^-0.6938*X* + 4.5608 (*R*^2^ = 0.8429). The optimal therapeutic window for HYW was identified at day 18 post-inoculation, with sustained efficacy observed from day 11 to day 23 (**Figure 4C**). These data provide critical temporal parameters for designing combination therapy regimens.

Further, Hypoxia probe (HP8) IHC was performed to visualize hypoxic regions. Quantitative analysis showed that HYW significantly reduced the area of hypoxic regions in 4T1 tumors (P < 0.001; **Figure 4D**), indicating improved oxygenation within the TME.

In conclusion, in the 4T1 TNBC, HYW promotes vascular function normalization by dual mechanisms: Inhibiting HIF-1α-mediated hypoxia signaling and enhancing tumor perfusion with a defined therapeutic window (days 11-23). These effects collectively reduce hypoxic regions, supporting the potential of HYW as a vascular normalizing agent.

### 3.5 HYW restores the balance between proangiogenic and antiangiogenic factors to facilitate “tumor vascular normalization”

To explore the molecular mechanism by which HYW facilitates “tumor vascular normalization”, ELISA was performed to quantify key angiogenic factors in 4T1 TNBC. Compared to the model group, HYW significantly reduced the protein levels of proangiogenic factors VEGFA (P < 0.01; **Figure 4E**) and bFGF (P < 0.01; **Figure 4E**). Conversely, the expression of antiangiogenic factors TSP-1 (P < 0.01; **Figure 4E**) and PF4 (P < 0.05; **Figure 4E**) was significantly elevated in the HYW group.

Heatmap of clusters further validated these findings. The heatmap revealed distinct clustering of the HYW group away from the model group, with proangiogenic factors (VEGFA, bFGF), hypoxia area clustered in the model group and antiangiogenic factors (TSP-1, PF4), CD31, α-SMA, VE-cadherin, α-SMA^+^/CD31^+^, vascular velocity enriched in the HYW group (**Figure 4F**). These data collectively indicate that HYW rebalances the angiogenic factor network, which may serve as a critical mechanism underlying its vascular normalization effects and subsequent improvements in tumor perfusion and hypoxia.

### 3.6 HYW enhances the efficacy of anti-PD-1 by promoting tumor vascular normalization

**Figure 5:**
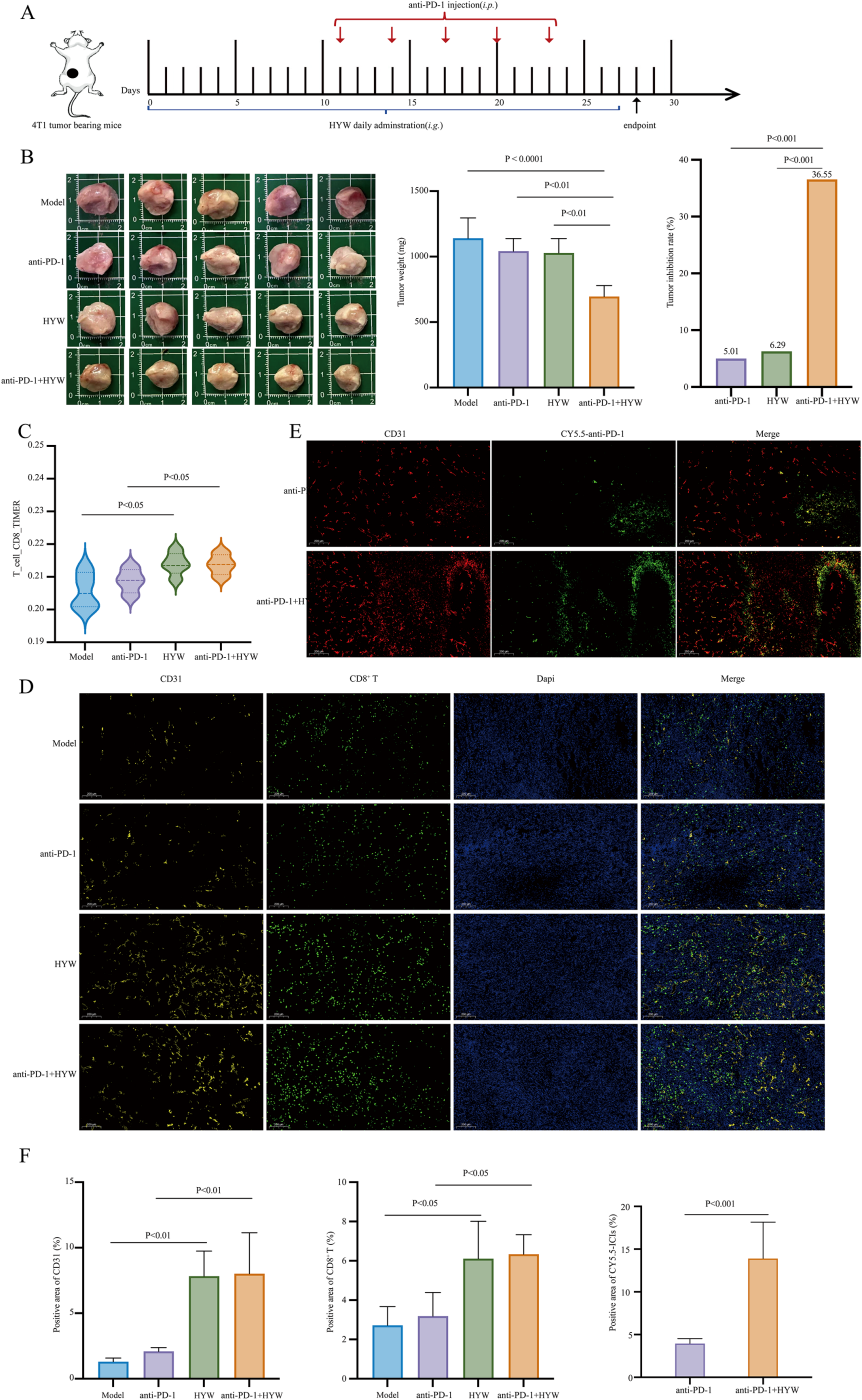
HYW enhances the efficacy of anti-PD-1 by promoting tumor vascular normalization. **(A)** Schematic plans of the animal experiment; **(B)** The general appearance of the breast tumors, tumor weight, and tumor inhibition rate of the experiment groups; **(C)** The relative infiltration ratio of CD8^+^ T cells were detected in model group, anti-PD-1 group, HYW group, and anti-PD-1 + HYW group by TIMER; **(D–F)** The expression of CD31, CD8^+^ T cells, Dapi and CY5.5-anti-PD-1 by IF.

To evaluate whether HYW enhances the efficacy of anti-PD-1, we established a 4T1 TNBC model and treated mice with a combination regimen of HYW and anti-PD-1, using the specified HYW intervention window (**Figure 5A**). The combination of HYW and anti-PD-1 significantly reduced breast tumor weight compared to anti-PD-1 alone (P < 0.01; **Figure 5B**), HYW alone (P < 0.01; **Figure 5B**), and model group (P < 0.0001; **Figure 5B**). The tumor inhibition rates were 36.55% (anti-PD-1 + HYW), 5.01% (anti-PD-1 alone), and 6.29% (HYW alone), respectively (**Figure 5B**), indicating a synergistic tumor-suppressive effect of the combination therapy. The tumor volume growth curve, which exhibited a similar trend, is provided in **Supplementary Figure 1**. TIMER analysis revealed that HYW monotherapy increased CD8^+^ T cell infiltration compared to the model group (P < 0.05), and this effect was further enhanced when combined with anti-PD-1 (anti-PD-1 + HYW vs. anti-PD-1 alone, P < 0.05; **Figure 5C**). IF staining confirmed that HYW significantly enhanced intratumoral CD8^+^ T cell infiltration (HYW vs. model group, P < 0.05; anti-PD-1 + HYW vs. anti-PD-1 alone, P < 0.05; **Figures 5D, F**) and enhanced the delivery of CY5.5-labeled anti-PD-1 (anti-PD-1 + HYW vs. anti-PD-1 alone, P < 0.001; **Figures 5E, F**). These findings support the hypothesis that HYW enhances anti-PD-1 efficacy by promoting “tumor vascular normalization”, thereby facilitating the infiltration of immune cells and the delivery of drugs into the tumor microenvironment.

### 3.7 HYW enhances anti-PD-1 efficacy in 4T1 TNBC by inhibiting PI3K-Akt-mTOR signaling, activating CD8^+^ T cells, and increasing effector cytokine release, inhibiting CD8^+^ T cell exhaustion, then remodeling the TIME

To investigate the underlying mechanisms by which HYW enhances anti-PD-1 efficacy, we performed transcriptome sequencing and functional validation experiments.

**Figure 6:**
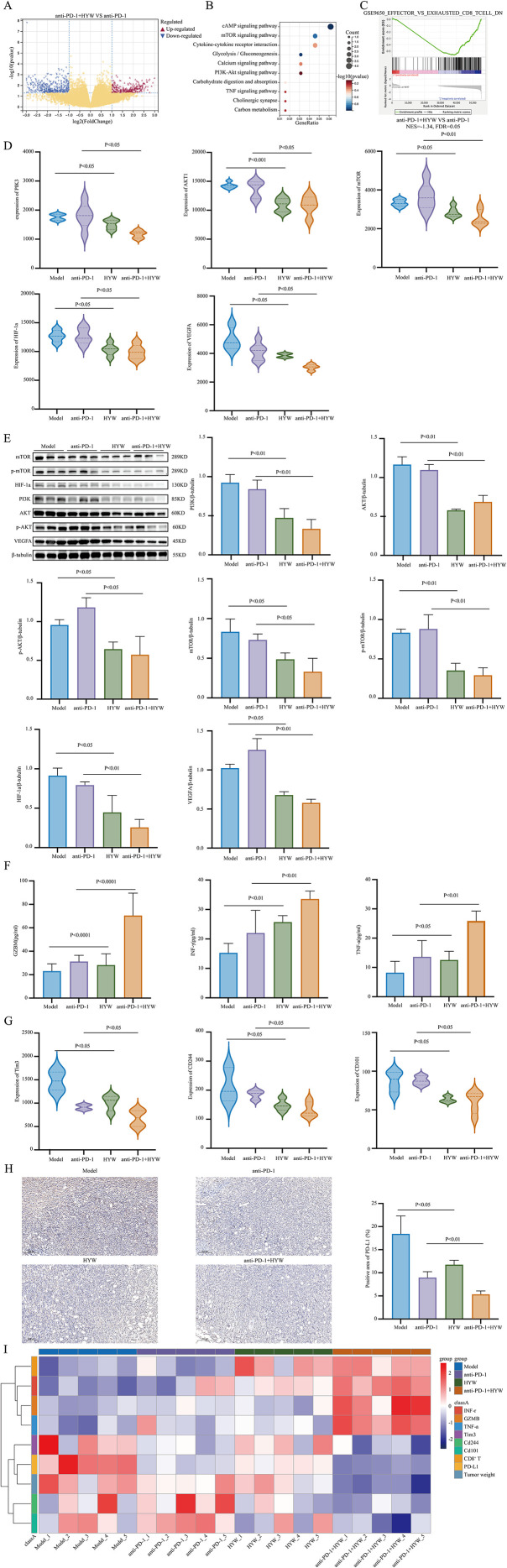
HYW enhances anti-PD-1 efficacy in 4T1 TNBC by inhibiting PI3K-Akt-mTOR signaling, activating CD8^+^ T cells, and increasing effector cytokine release, inhibiting CD8^+^ T cell exhaustion, then remodeling the tumor immune microenvironment. **(A)** Volcano plot of the DEGs between anti-PD-1 + HYW group and anti-PD-1 group; **(B)** KEGG pathway enrichment analysis; **(C)** The analysis of “EFFECTOR_VS_EXHAUSTED_CD8_T CELL” pathways between anti-PD-1 + HYW and anti-PD-1 by GSEA; **(D)** The mRNA expression of PI3K, Akt, mTOR, HIF-1α and VEGFA were detected in model group, anti-PD-1 group, HYW group, and anti-PD-1 + HYW group by transcriptome sequencing; **(E)** The protein expression of mTOR, p-mTOR, HIF-1α, PI3K, Akt, p-Akt, VEGFA were detected in model group, anti-PD-1 group, HYW group, and anti-PD-1 + HYW group by western blot; **(F)** The protein expression of GZMB, IFN-γ, and TNF-α in plasma samples were detected in model group, anti-PD-1 group, HYW group, and anti-PD-1 + HYW group by ELISA; **(G)** The mRNA expression of TIM3, CD244, and CD101 were detected in model group, anti-PD-1 group, HYW group, and anti-PD-1 + HYW group by transcriptome sequencing; **(H)** The protein expression of PD-L1 were detected in model group, anti-PD-1 group, HYW group, and anti-PD-1 + HYW group by IHC; **(I)** Heatmap of clusters.

Firstly, transcriptome analysis identifies key pathways: Comparative transcriptome sequencing of tumors from the anti-PD-1 + HYW vs anti-PD-1 alone groups identified 746 DEGs, including 302 upregulated and 444 downregulated genes (**Figure 6A**). KEGG pathway enrichment analysis revealed 198 enriched pathways, with the top 10 including the PI3K-Akt signaling pathway, mTOR signaling pathway, and cytokine-cytokine receptor interactions (**Figure 6B**). Based on prior research and literature, the PI3K-Akt-mTOR pathway was selected for further validation. Additionally, GSEA showed significant enrichment of the “EFFECTOR_VS_EXHAUSTED_CD8_T_CELL” pathway in the anti-PD-1 + HYW group (**Figure 6C**), suggesting that HYW may inhibit CD8^+^ T cell exhaustion.

Next, HYW inhibits PI3K-Akt-mTOR signaling and downstream effectors: Transcriptome data showed that HYW monotherapy significantly reduced mRNA expression of PI3K, Akt, mTOR, HIF-1α, and VEGFA compared to the model group (PI3K: P < 0.05; Akt: P < 0.001; mTOR: P < 0.05; HIF-1α: P < 0.05; VEGFA: P < 0.05; **Figure 6D**), and these effects were further enhanced in the anti-PD-1 + HYW group compared to anti-PD-1 alone (PI3K: P < 0.05; Akt: P < 0.05; mTOR: P < 0.01; HIF-1α: P < 0.05; VEGFA: P < 0.05; **Figure 6D**). Western blot analysis confirmed that HYW monotherapy significantly reduced protein levels of key PI3K-Akt-mTOR pathway components, as well as downstream HIF-1α and VEGFA (PI3K: P < 0.01; Akt: P < 0.01; p- Akt: P < 0.05; mTOR: P < 0.05; p-mTOR: P < 0.01; HIF-1α: P < 0.05; VEGFA: P < 0.05; **Figure 6E**), and these effects were further enhanced in the anti-PD-1 + HYW group compared to anti-PD-1 alone (PI3K: P < 0.01; Akt: P < 0.01; p- Akt: P < 0.05; mTOR: P < 0.05; p-mTOR: P < 0.01; HIF-1α: P < 0.01; VEGFA: P < 0.01; **Figure 6E**). Which was consistent with the transcriptome results.

Furthermore, HYW promotes CD8^+^ T cell activation: ELISA assays of plasma samples showed that HYW monotherapy significantly increased secretion of GZMB, IFN-γ, and TNF-α compared to the model group (GZMB: P < 0.0001; IFN-γ: P < 0.01; TNF-α: P < 0.05; **Figure 6F**), and these effects were further enhanced in the anti-PD-1 + HYW group compared to anti-PD-1 alone (GZMB: P < 0.0001; IFN-γ: P < 0.01; TNF-α: P < 0.01; **Figure 6F**), indicating enhanced CD8^+^ T cell activity.

Additionally, HYW inhibits CD8^+^ T cell exhaustion and reduces PD-L1 expression: Transcriptome analysis demonstrated that HYW monotherapy downregulated mRNA expression of the exhaustion markers Tim3, CD244, and CD101 compared to the model group (all P < 0.05; **Figure 6G**), with further reductions in the anti-PD-1 + HYW group vs. anti-PD-1 alone (all P < 0.05; **Figure 6G**). Additionally, HYW significantly decreased PD-L1 protein expression compared to the model group (P < 0.05; **Figure 6H**), and this effect was more pronounced in the anti-PD-1 + HYW group vs. anti-PD-1 alone (P < 0.01; **Figure 6H**).

Finally, a heatmap of gene clusters further confirmed that HYW enhances anti-PD-1 efficacy by activating CD8^+^ T cells, and increasing effector cytokines release, inhibiting CD8^+^ T cell exhaustion, then remodeling the TIME (improving the immunosuppressive TME into an immunesupportive TME). (**Figure 6I**).

## 4 Discussion

TNBC remains a clinical challenge due to its aggressive phenotype and limited responsiveness to conventional therapies (1, 2). Despite the breakthrough of ICIs and the recognition of TNBC as a preferred subtype for ICIs treatment, most patients exhibit limited responses to ICIs alone, with an overall response rate of less than 20% (21). This limitation is primarily attributed to the immunosuppressive TME, particularly aberrant angiogenesis and hypoxia. In this study, we demonstrate that HYW, a TCM formulation, enhances anti-PD-1 efficacy in TNBC by normalizing tumor vasculature and remodeling the TIME. Our findings provide a novel mechanistic basis for combining TCM with anti-PD-1 to enhance anti-PD-1 efficacy in TNBC.

TCM has long been recognized for its clinical advantages in cancer treatment, including minimal adverse effects, favorable therapeutic outcomes, and prolonged patient survival (51). Notably, a core TCM principle in oncology is addressing “Qi stagnation and blood stasis”, which is recognized as a pivotal pathogenic factor in breast cancer pathogenesis (52). The pathological state of “Qi stagnation and blood stasis” frequently induces impaired blood circulation and obstruction of tissue fluid drainage within the TME, thereby creating a localized hypoxic environment that promotes angiogenesis and subsequent tumor progression (53). Consequently, the therapeutic strategy of “activating blood circulation and resolving stasis”-as embodied by the “replenishing Qi, activating blood circulation, and detoxifying” (YQHXJD) principle-serves as a cornerstone of TCM oncological practice (54–57). Mechanistically, blood-activating and stasis-resolving formulas have been shown to ameliorate TME hypoxia by improving microcirculatory perfusion (58). As a representative YQHXJD formulation, HYW (37–41) has exhibited efficacy in inhibiting tumor growth, enhancing patient quality of life, and suppressing metastasis in murine models of 4T1 TNBC and Lewis lung carcinoma. Furthermore, HYW augments chemotherapy efficacy by improving intratumoral drug delivery, a mechanism aligned with the “tumor vascular normalization” theory. This prior evidence, combined with TNBC’s high angiogenic activity, provided the rationale to investigate HYW as a potential modulator of ICIs responsiveness.

Tumor angiogenesis is a hallmark of cancer progression, and preclinical and clinical studies have confirmed that tumor vascular normalization aims to normalize aberrant tumor vasculature, restore stable blood flow and perfusion, and thereby enhance intratumoral delivery of ICIs and immune cells, converting the immunosuppressive TME into an immune-supportive TME (30–35). The intimate interplay between tumor progression and angiogenesis has been well established over decades of research (59, 60), with substantial evidence confirming that solid tumors exhibit an “angiogenesis-dependent” phenotype (56, 57, 61). Among the key mediators of this process, VEGFA stands as the most potent inducer of tumor angiogenesis (61–63), whose overexpression in TNBC (confirmed by our TCGA analysis) drives vascular abnormalities and hypoxia, and strategies aimed at restoring tumor vascular normalization have predominantly focused on inhibiting VEGFA signaling (64–66).

Our TCGA analysis confirmed that VEGFA, a key proangiogenic factor, is overexpressed in TNBC and correlates with angiogenic pathway activation, consistent with previous reports highlighting pronounced vascular structural and functional abnormalities in TNBC compared to other subtypes (29). Network pharmacology predicted that HYW targets VEGFA and modulates the PI3K-Akt/HIF-1 signaling axis, as demonstrated by transcriptomic and functional studies. Moreover, the precise mechanism by which HYW regulates VEGFA remains to be elucidated through molecular biology experiments or rescue experiments using VEGFA agonists.

“Tumor vascular normalization” refers to the process by which tumor vessels acquire normalized structural and functional phenotypes (67). Vascular structural normalization is characterized by morphological remodeling of the tumor vascular network, with key features including: ECs lining (68): ECs form a continuous, intact inner layer of blood vessels, contributing to enhanced vascular barrier function; PCs coverage (69): PCs are recruited to ensheath ECs in microvessels (e.g., capillaries, small postcapillary venules, and terminal arterioles) and integrate into the vascular basement membrane; Reduction of pathological phenotypes: Significant decreases in tumor vascular density and tortuosity.

Hypoxia is a hallmark of most solid tumors and a common trait of TME induced by vascular structural abnormalities (70–73). In breast cancer, hypoxia is more pronounced in TNBC than in other breast cancer subtypes (29, 74). To better recapitulate the *in vivo* microenvironment, we performed a series of functional assays targeting key steps in vascular endothelial cell biology under normal oxygen/hypoxic condition to evaluate the vascular structural normalization potential of HYW *in vitro* experiments. HYW exerted dual, oxygen-dependent effects on EC functions: under normal condition, it inhibited proliferation, tube formation, migration, and invasion-suppressing *de novo* angiogenesis; under hypoxia condition, it potentiated anti-angiogenic effects on proliferation/tube formation while promoting EC migration/invasion, a phenotype consistent with vascular remodeling. This biphasic regulation aligns with the “vascular normalization window” theory (34).

Mechanistically, under physiological conditions, the balance between proangiogenic factors-including VEGF, bFGF, PF4 and TSP-1 is critical for maintaining vascular homeostasis (27, 28). In the TME, tumor and stromal cells secrete multiple proangiogenic factors, disrupting this balance and driving the development of structurally abnormal and functionally impaired blood vessels, and this pathological process is further exacerbated under hypoxic conditions (27, 28). Importantly, restoring the balance between proangiogenic and antiangiogenic factors has been shown to induce “tumor vascular normalization” (75). In this study, HYW reduced proangiogenic factors VEGFA and bFGF, while increasing antiangiogenic factors TSP-1 and PF4, rebalancing the angiogenic network. Transmission electron microscopy and IF further confirmed structural normalization, with increased pericyte coverage (α-SMA), improved endothelial junction integrity (VE-cadherin) and tumor vascular maturity (α-SMA^+^/CD31^+^ ratio) (76).

Functionally, vascular functional normalization (77) is characterized by enhancing the delivery of anticancer agents (chemotherapy, targeted therapy, ICIs, and so on) and immune cells (CD8^+^ T cells, NK cells, macrophages, and so on) into the tumor parenchyma, while concomitantly alleviating intratumoral hypoxia. Preclinical and clinical studies have confirmed that hypoxia drives the formation of an immunosuppressive tumor microenvironment, promotes tumor immune evasion, and impairs ICIs-mediated antitumor immune responses (78–87). These findings suggest that mitigating tumor hypoxia may partially enhance the therapeutic efficacy of ICIs in TNBC.

In this study, HYW alleviated tumor hypoxia by downregulating HIF-1α and enhancing perfusion, as evidenced by ultrasound imaging and hypoxyprobe staining. This is critical, as hypoxia drives immune suppression by upregulating PD-L1 and limiting CD8^+^ T cell infiltration (35). By normalizing vasculature, HYW creates a permissive TME for anti-PD-1 and immune cells-a finding supported by increased intratumoral delivery of CY5.5-labeled anti-PD-1 and CD8^+^ T cells in the HYW + anti-PD-1 group compared to the anti-PD-1 group.

During pharmacological anti-angiogenic therapy, a transient “vascular normalization window” (67) typically emerges, characterized by structurally and functionally normalized tumor vessels. However, this window is highly transient and challenging to capture, with its duration and dynamics potentially varying by tumor type (88–91). Notably, initiation of ICIs within the “vascular normalization window” may significantly enhance antitumor efficacy. In 4T1 TNBC model, the cubic regression model fitted for HYW-induced vascular normalization was expressed as: *Y*=-0.0011*X*3+0.049*X*2-0.6938*X*+4.5608 (*R2*=0.8429), indicating HYW-induced tumor vascular normalization also exhibited a time-window characteristic, with the optimal therapeutic window identified at day 18 post-tumor inoculation and a sustained efficacy period from day 11 to day 23. While these data identify a temporal window for HYW-induced vascular normalization in the 4T1 mouse TNBC model, it is essential to recognize that these findings are preclinical. The presence, optimal timing, and duration of a similar “vascular normalization window” in humans, especially for combining with anti-PD-1 therapy, require validation through future translational and clinical research.

As we all know, the efficacy of ICIs depends on the infiltration and activation of CD8^+^ T cells. In our 4T1 model, HYW monotherapy increased CD8^+^ T cell infiltration, and combination with anti-PD-1 further enhanced this effect, leading to synergistic tumor growth inhibition (tumor inhibition rate: 36.55% vs. 5.01% for anti-PD-1 alone). Transcriptomic analysis revealed that HYW downregulates the PI3K-Akt-mTOR pathway, a key driver of tumor growth and immune suppression. Inhibition of this pathway reduced HIF-1α and VEGFA expression, breaking the hypoxia-angiogenesis-immunosuppression cycle. Moreover, HYW promoted CD8^+^ T cell activation by increasing effector cytokines (GZMB, IFN-γ, TNF-α) and reducing exhaustion markers (Tim3, CD244, CD101). This is consistent with KEGG enrichment of T cell receptor signaling and PD-L1/PD-1 checkpoint pathways. Notably, HYW decreased PD-L1 expression in tumors, potentially sensitizing cancer cells to ICIs. Together, these data suggest HYW transforms the TME from immunosuppressive to immunesupportive, amplifying ICIs’ antitumor activity.

Our study provides preclinical evidence for combining HYW with anti-PD-1, addressing a critical unmet need in TNBC treatment. The optimal therapeutic window for HYW (days 11–23 post inoculation) identified by ultrasound imaging offers practical guidance for clinical scheduling, ensuring maximal vascular normalization before ICIs administration. The oxygen-dependent regulation of EC functions by HYW highlights its potential to adapt to the dynamic TME, distinguishing it from conventional anti-angiogenic agents (bevacizumab) that often cause excessive vascular pruning. Additionally, the identification of 109 chemical constituents in HYW (via LC-MS) provides a basis for isolating active compounds (targeting VEGFA or PI3K-Akt) for future drug development.

## 5 Conclusion

**Figure 7:**
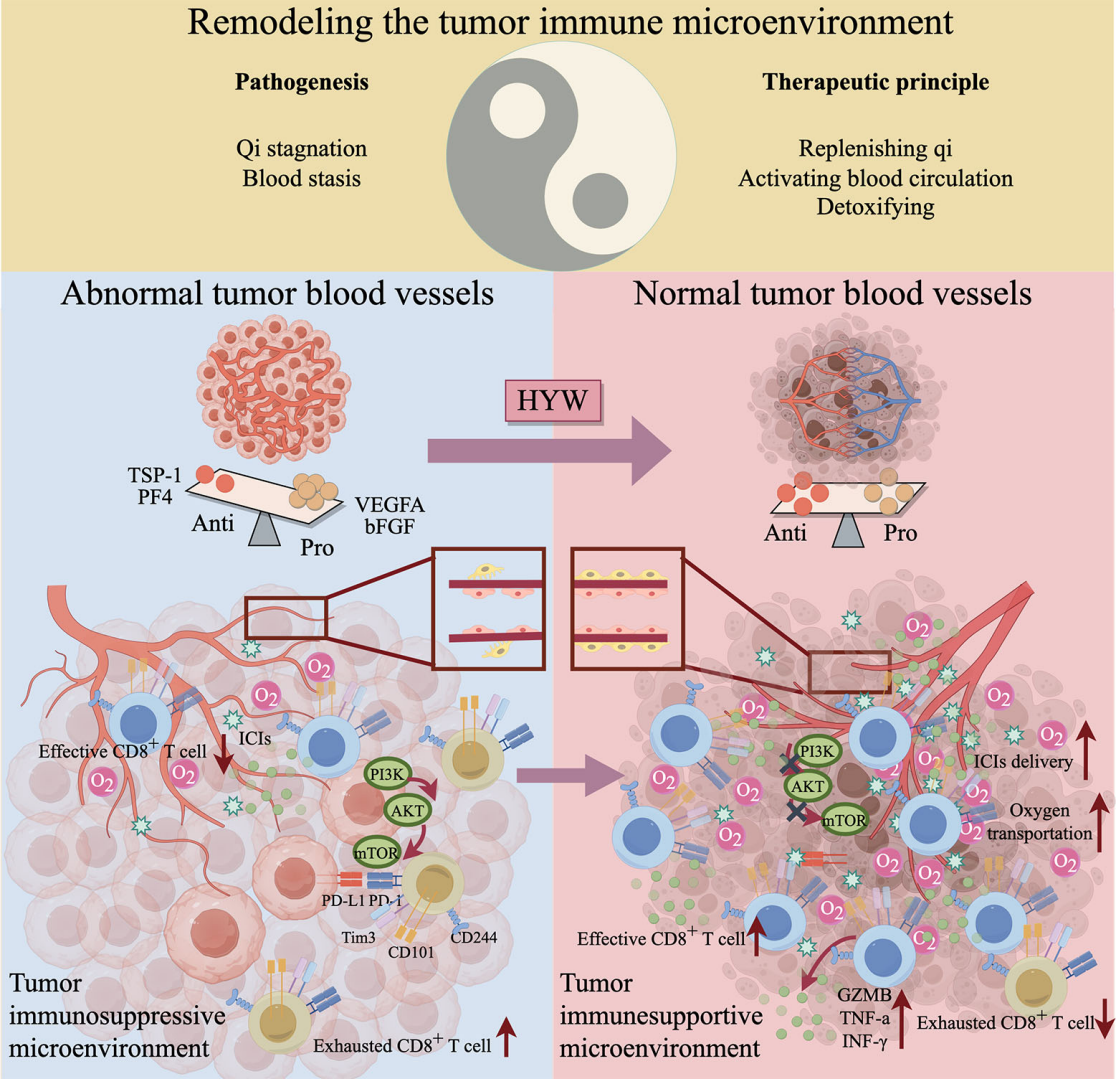
Mechanism of HYW in enhancing the efficacy of anti-PD-1 in the treatment of TNBC (By Figdraw). HYW enhances anti-PD-1 efficacy in TNBC by normalizing tumor vasculature, rebalancing angiogenic factors, and remodeling the TIME to promote CD8^+^ T cell infiltration, activation, and inhibit CD8^+^ T cell exhaustion. Drawn by Figdraw and copyright Code: ITPYYc22c7.

HYW enhances anti-PD-1 efficacy in TNBC by normalizing tumor vasculature, rebalancing angiogenic factors, and remodeling the TIME to promote CD8+ T cell infiltration, activation, and inhibit CD8+ T cell exhaustion (**Figure 7**). These findings support combining HYW with anti-PD-1 as a novel therapeutic strategy for TNBC, addressing the critical unmet need for improved ICIs responsiveness.

## Data Availability

The datasets presented in this study can be found in online repositories. The names of the repository/repositories and accession number(s) can be found in the article/**Supplementary Material**.

## Glossary

bFGF: Basic Fibroblast Growth Factor
CSCO: Chinese Society Of Clinical Oncology
DEGs: Differentially Expressed Genes
ECL: Enhanced Chemiluminescence
ER: Estrogen Receptor
FDR: False Discovery Rate
FDA: Food And Drug Administration
GO: Gene Ontology
GZMB: Granzyme B
HESI: Heated Electrospray Ionization
HYW: Huayu Wan
HER2: Human Epidermal Growth Factor Receptor 2
HUVECs: Human Umbilical Vascular Endothelial Cells
ICIs: Immune Checkpoint Inhibitors
IOBR: Immune Oncology Biomarker Research
IFN-γ: Interferon-Gamma
LC-MS: Liquid Chromatography-Mass Spectrometry
KEGG: Kyoto Encyclopedia Of Genes And Genomes
ANOVA: One-Way Analysis Of Variance
OD: Optical Density
ORR: Overall Response Rate
PB: Phosphate Buffer
PF4: Platelet Factor 4
PR: Progesterone Receptor
PPI: Protein-Protein Interaction
SD: Standard Deviation
TCGA: The Cancer Genome Atlas
DAVID: The Database For Annotation, Visualization And Integrated Discovery
TSP-1: Thrombospondin-1
TCM: Traditional Chinese Medicine
TPM: Transcripts Per Million
TNBC: Triple-Negative Breast Cancer
TIME: Tumor Immune Microenvironment
TILs: Tumor Infiltrating Lymphocytes
TME: Tumor Microenvironment
TMB: Tumor Mutational Burden
TNF-a: Tumor Necrosis Factor-Alpha
VEGFA: Vascular Endothelial Growth Factor A
YQHXJD: Yiqi Huoxue Jiedu
