# Supplementary figures and images for: Huayu Wan enhances immune checkpoint inhibitor efficacy in triple-negative breast cancer by normalizing tumor vasculature and remodeling the tumor immune microenvironment
Source: Front Immunol. 2026 May 19;17:1824721. doi: 10.3389/fimmu.2026.1824721 (PMC13227399; doi:10.3389/fimmu.2026.1824721)

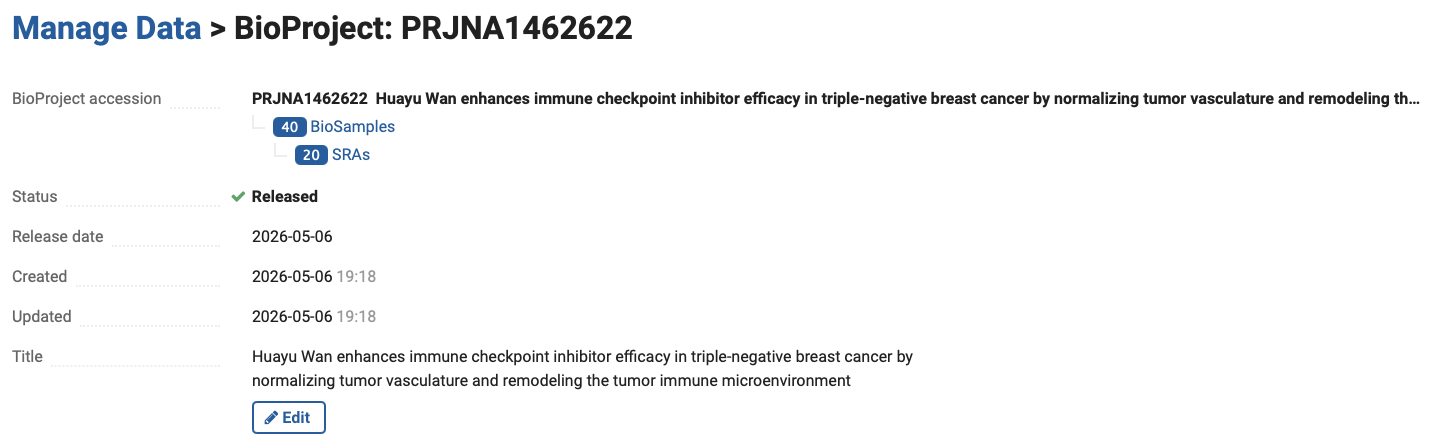

Supplement: Supplementary file 1 [file SupplementaryFile1.png]

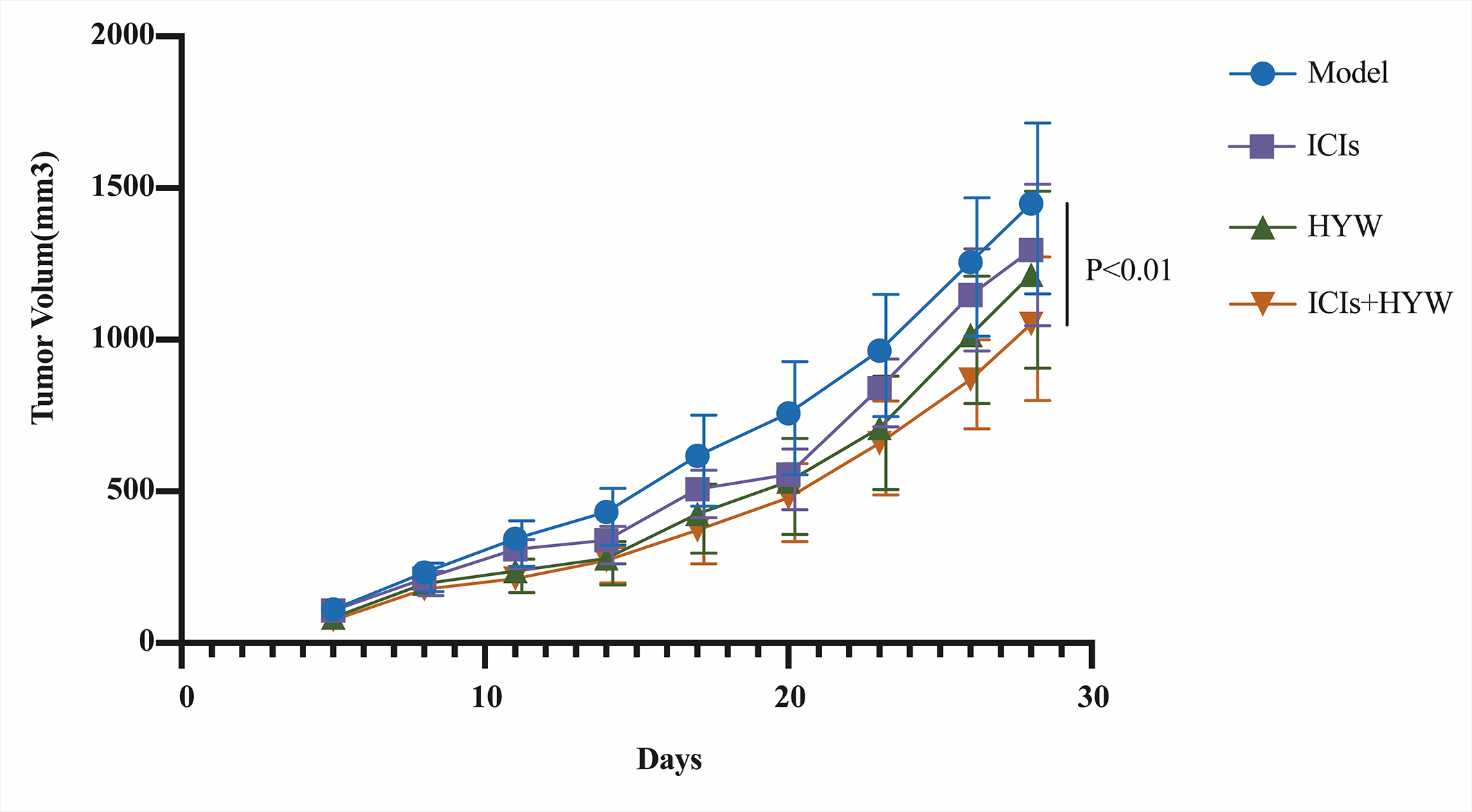

Supplement: Supplementary file 2 [file Image1.tif]

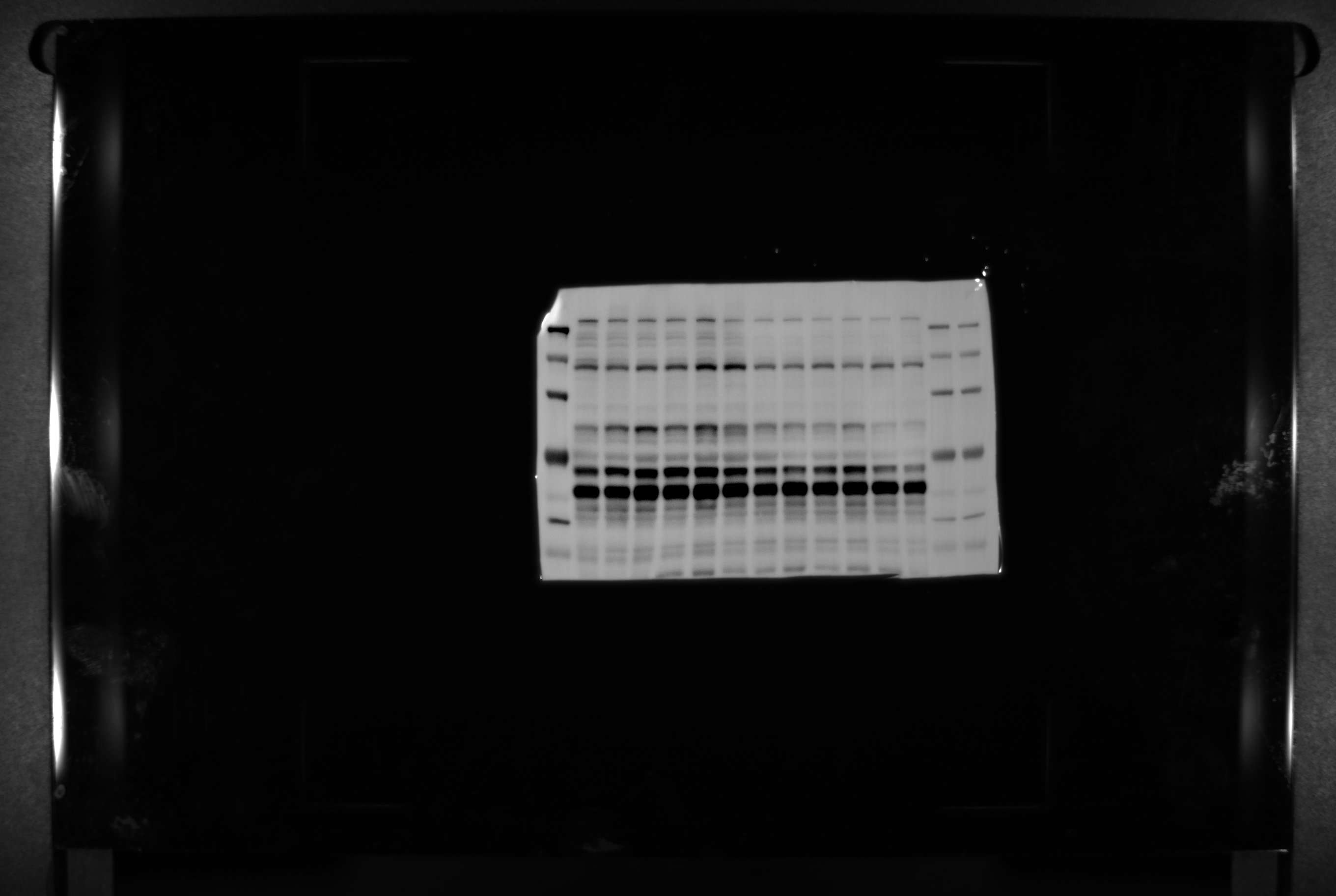

Supplement: Supplementary file 3 [file Image2.tiff]

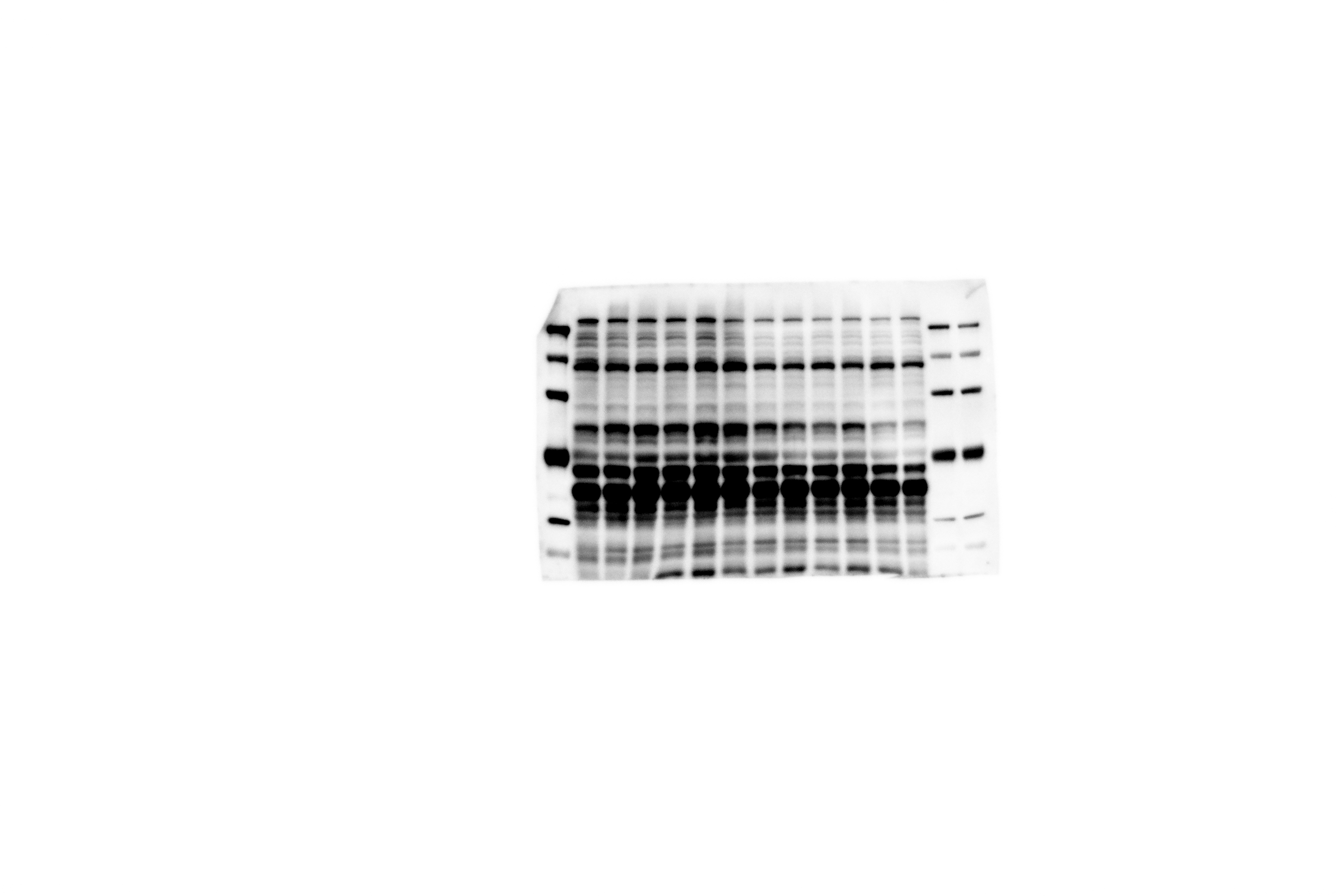

Supplement: Supplementary file 4 [file Image3.tiff]

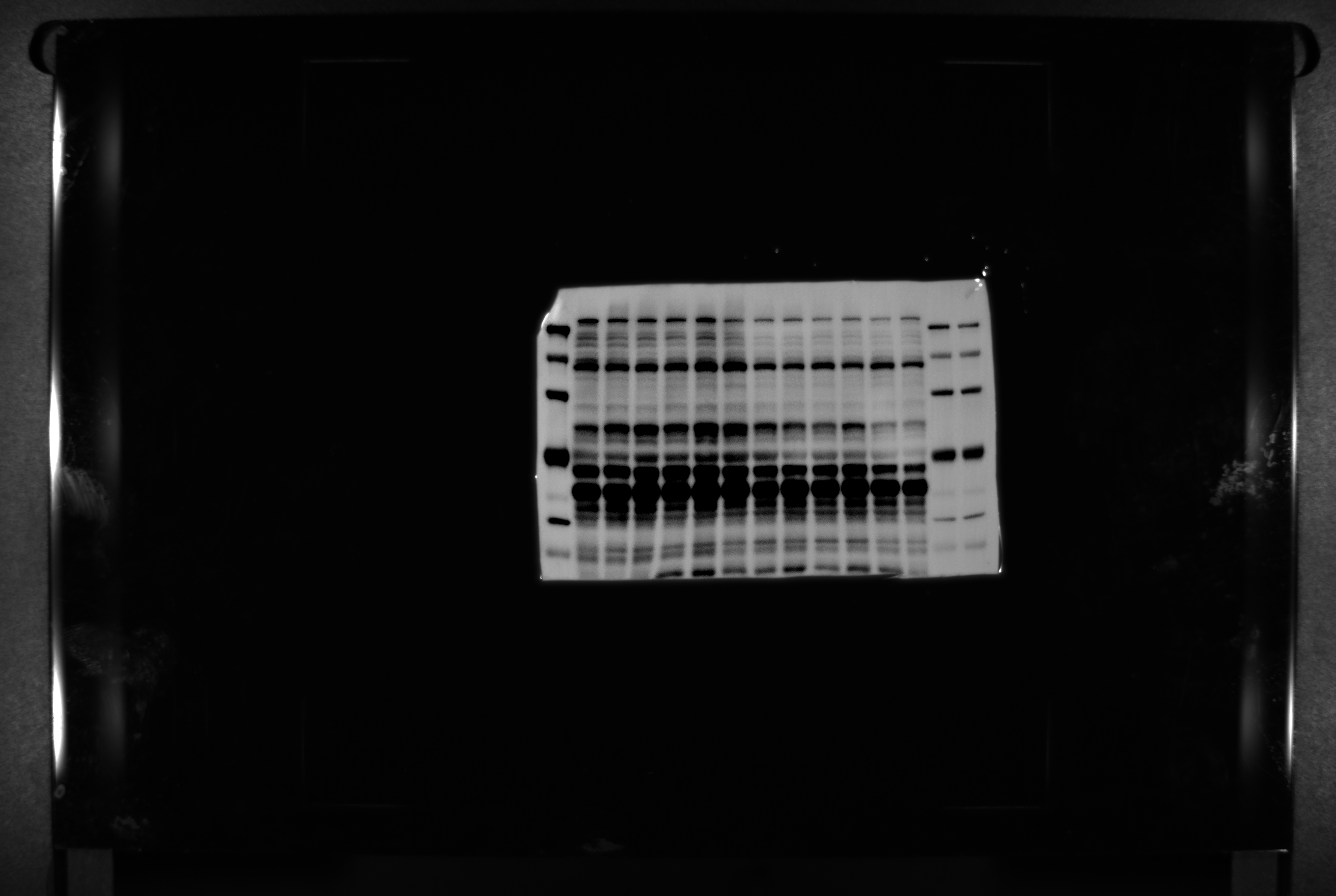

Supplement: Supplementary file 5 [file Image4.tiff]

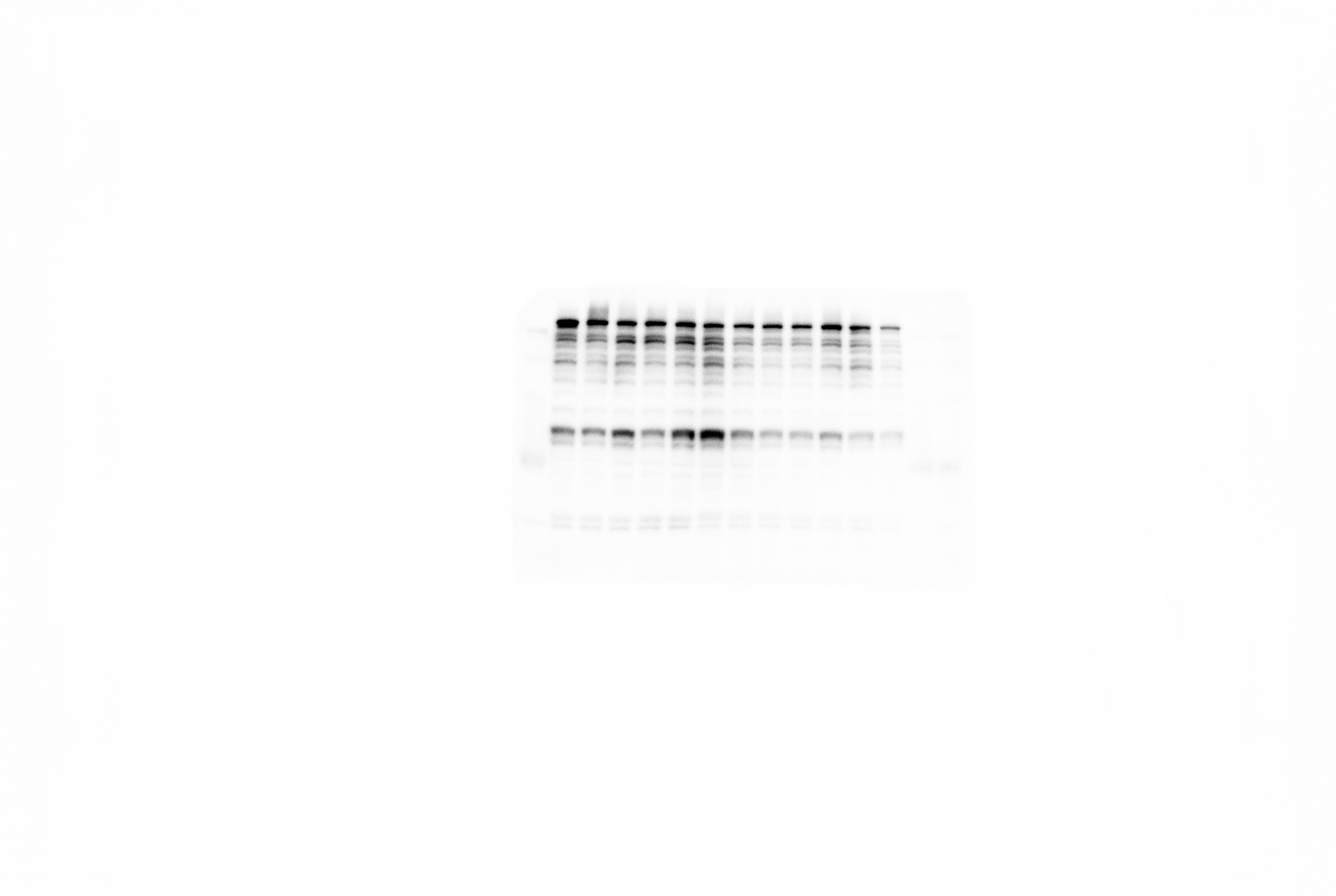

Supplement: Supplementary file 6 [file Image5.tiff]

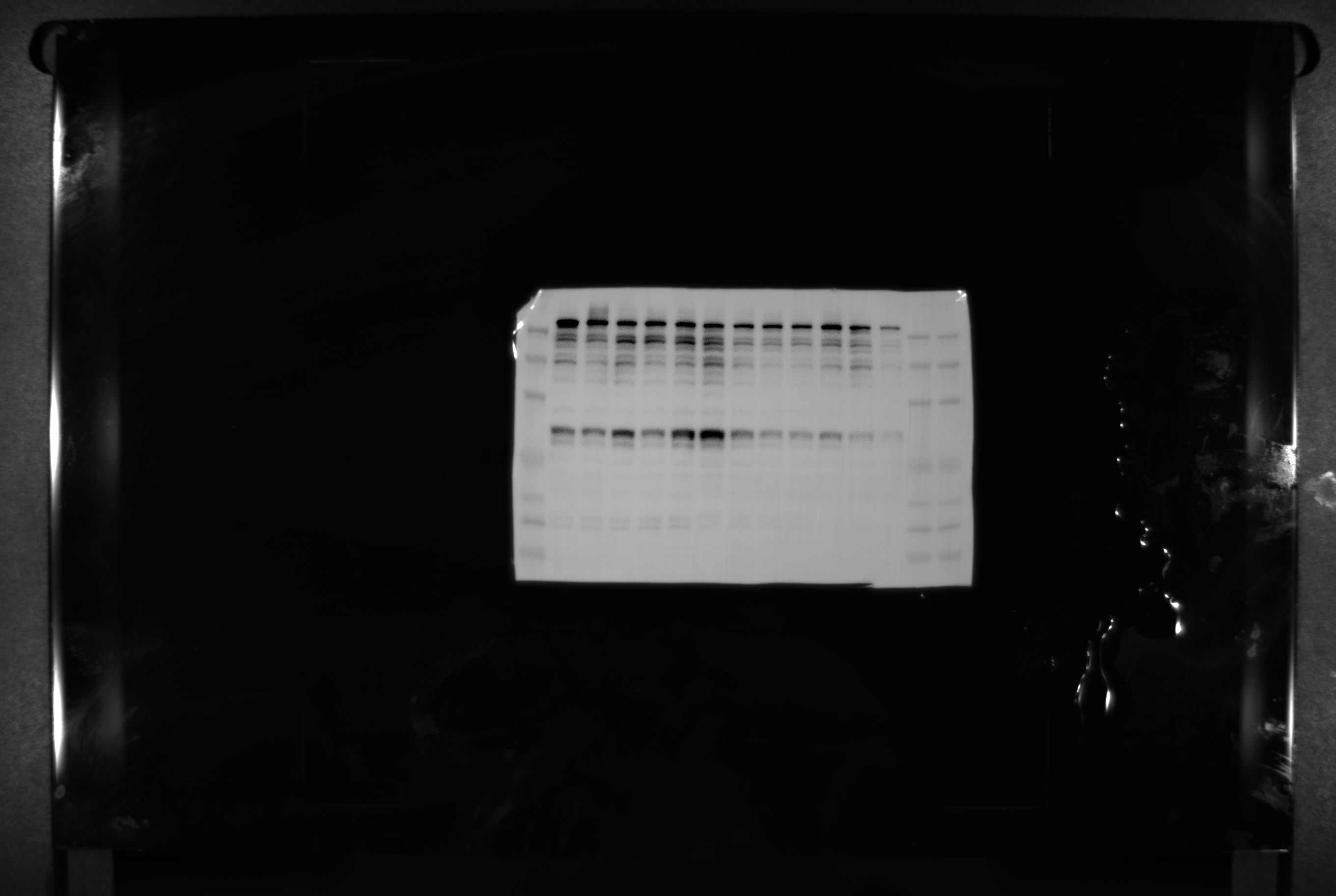

Supplement: Supplementary file 7 [file Image6.tiff]

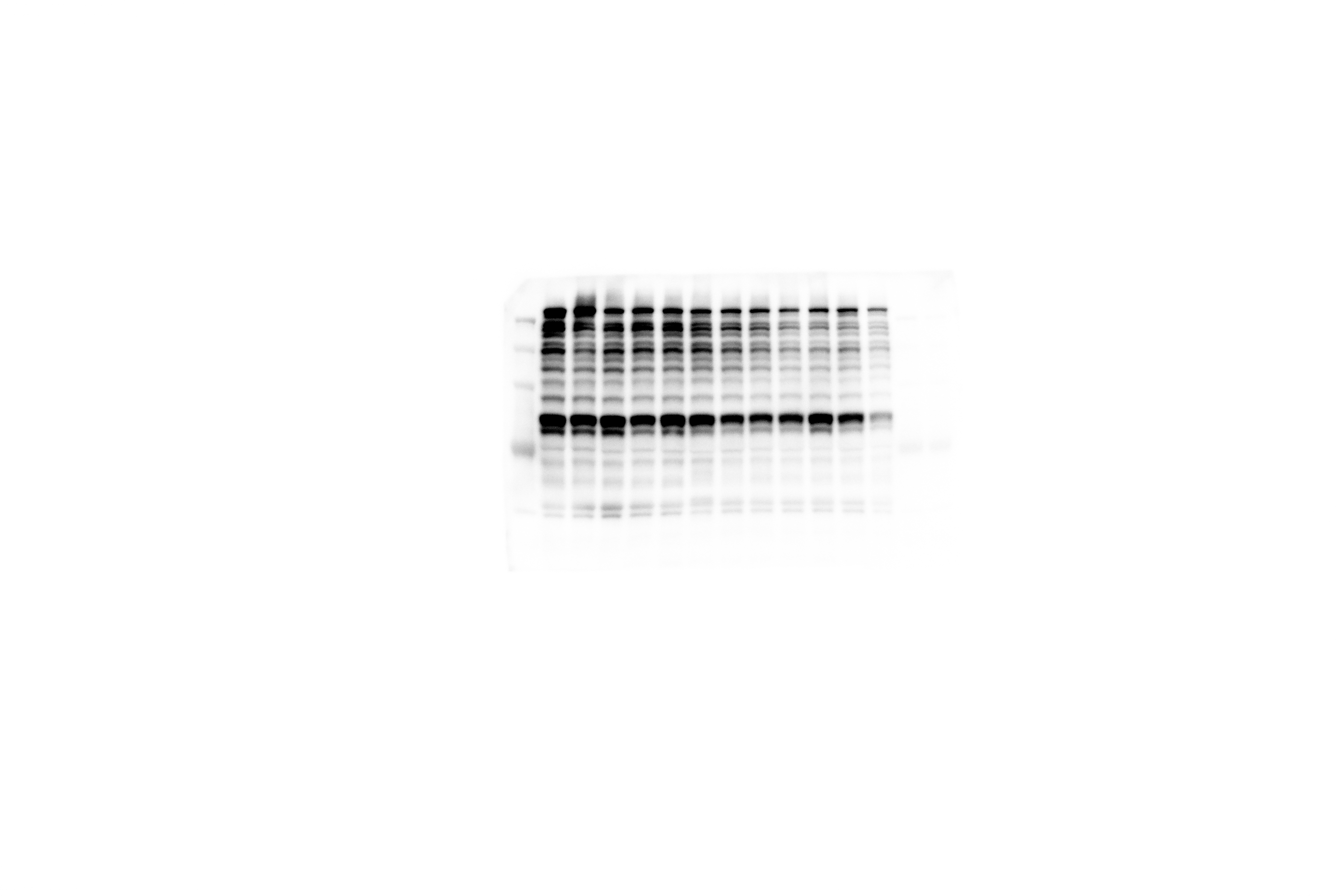

Supplement: Supplementary file 8 [file Image7.tiff]

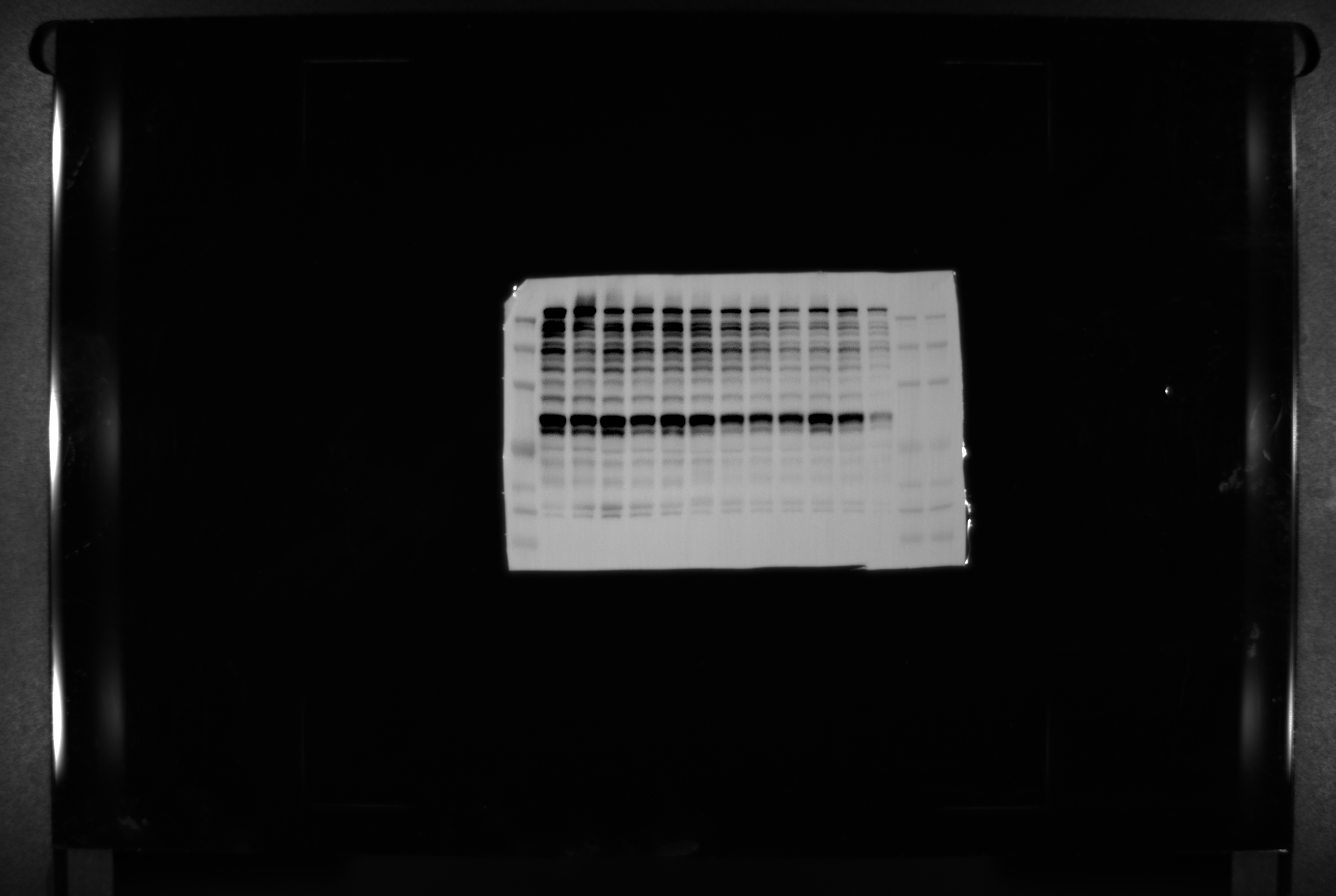

Supplement: Supplementary file 9 [file Image8.tiff]

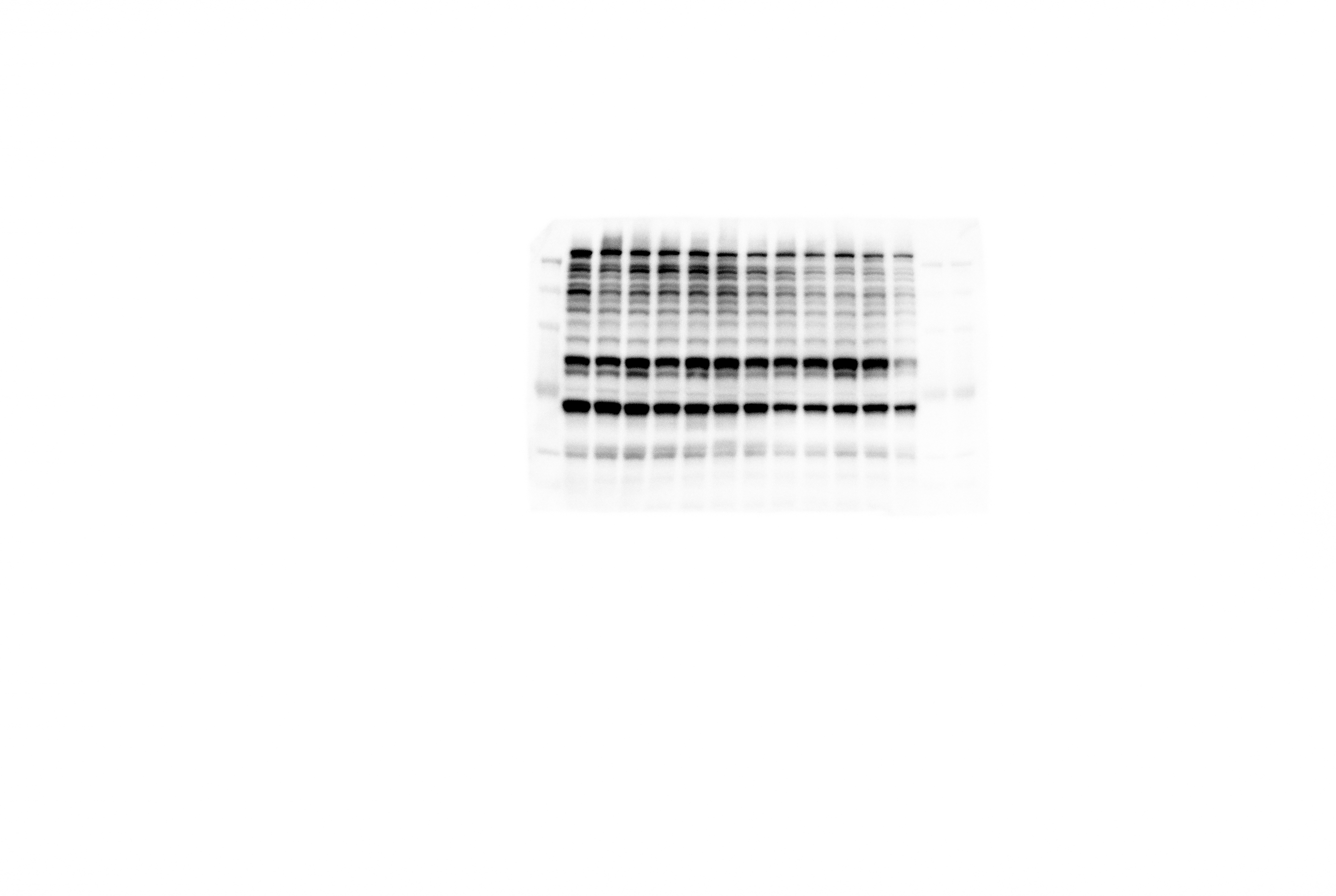

Supplement: Supplementary file 10 [file Image9.tiff]

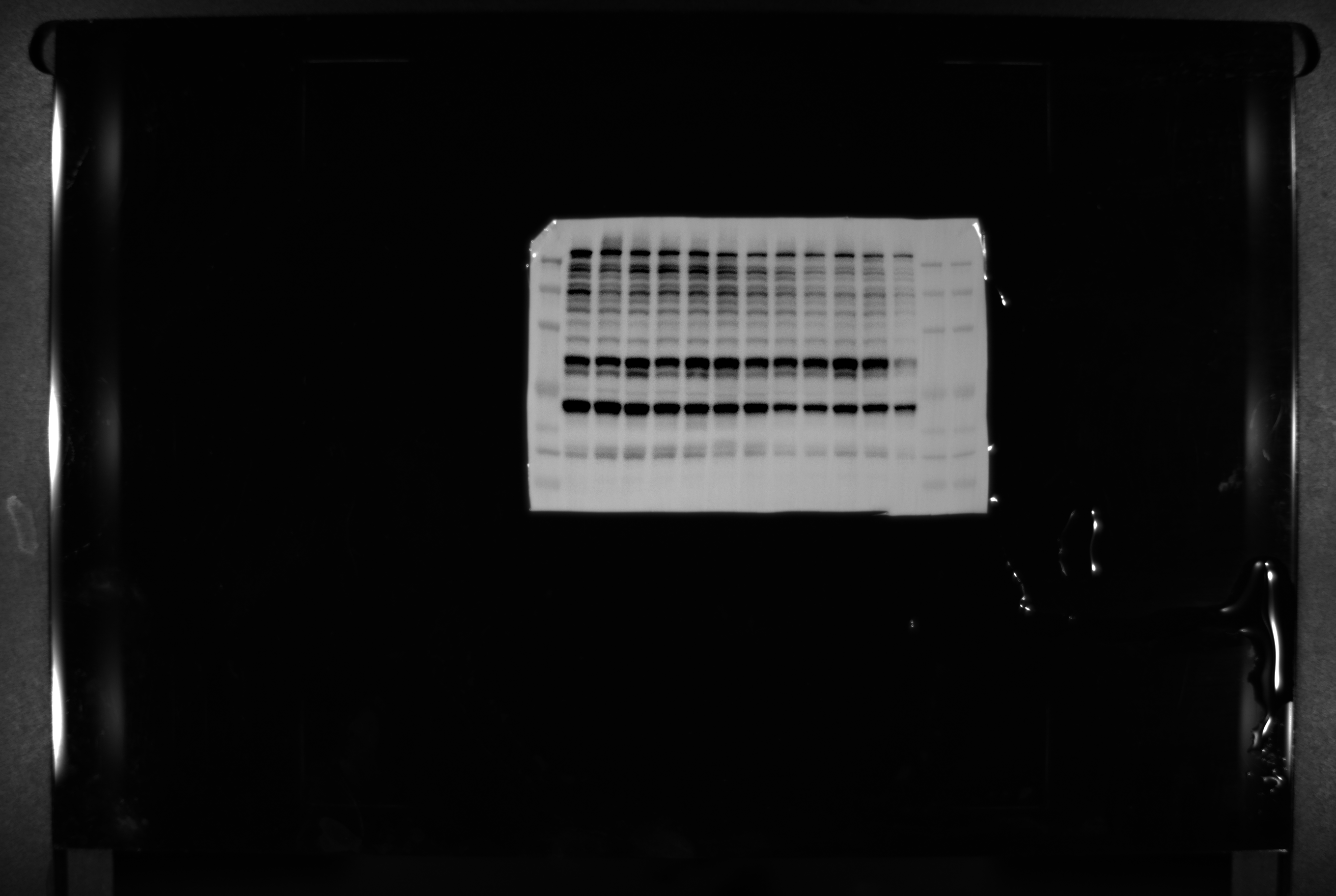

Supplement: Supplementary file 11 [file Image10.tiff]

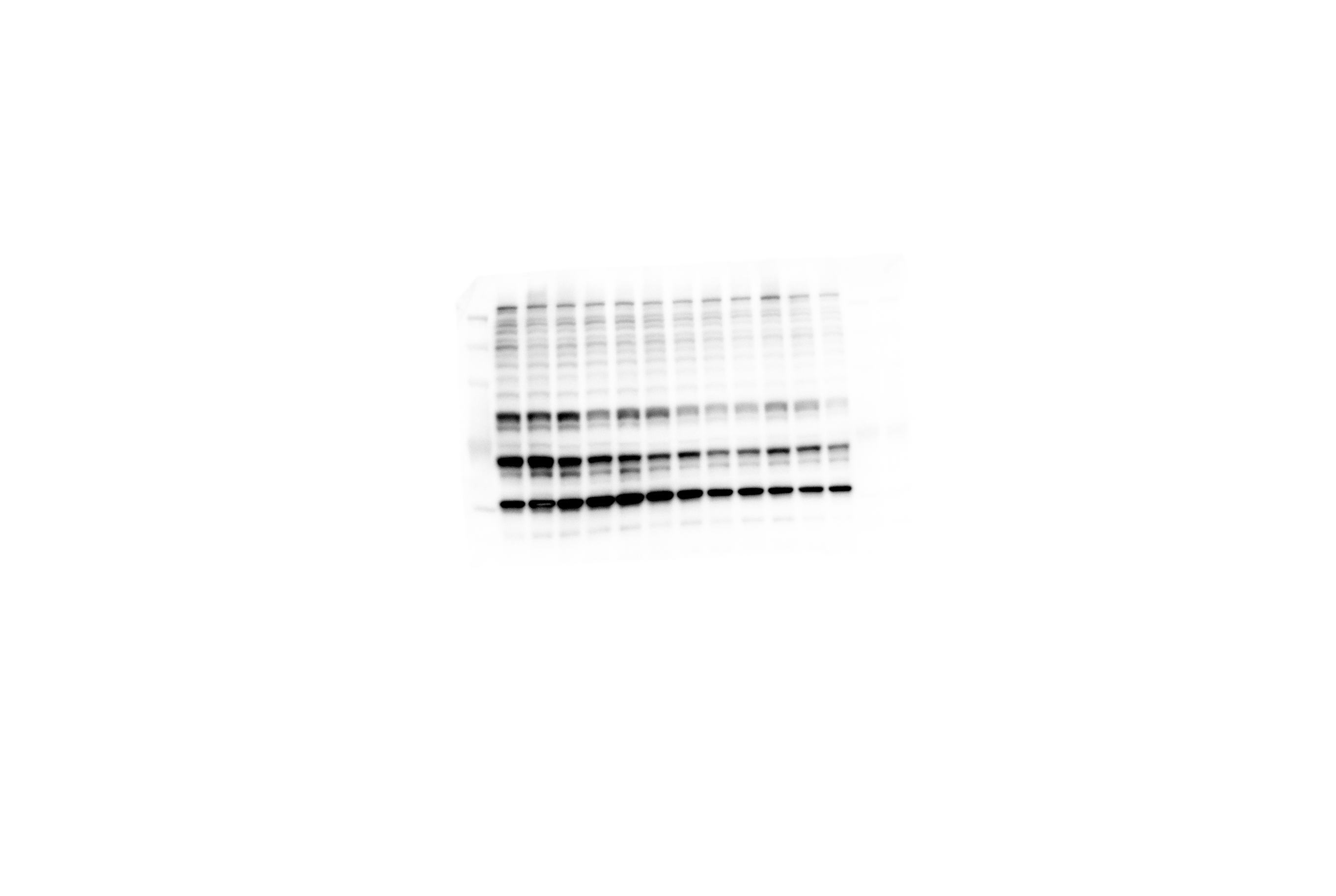

Supplement: Supplementary file 12 [file Image11.tiff]

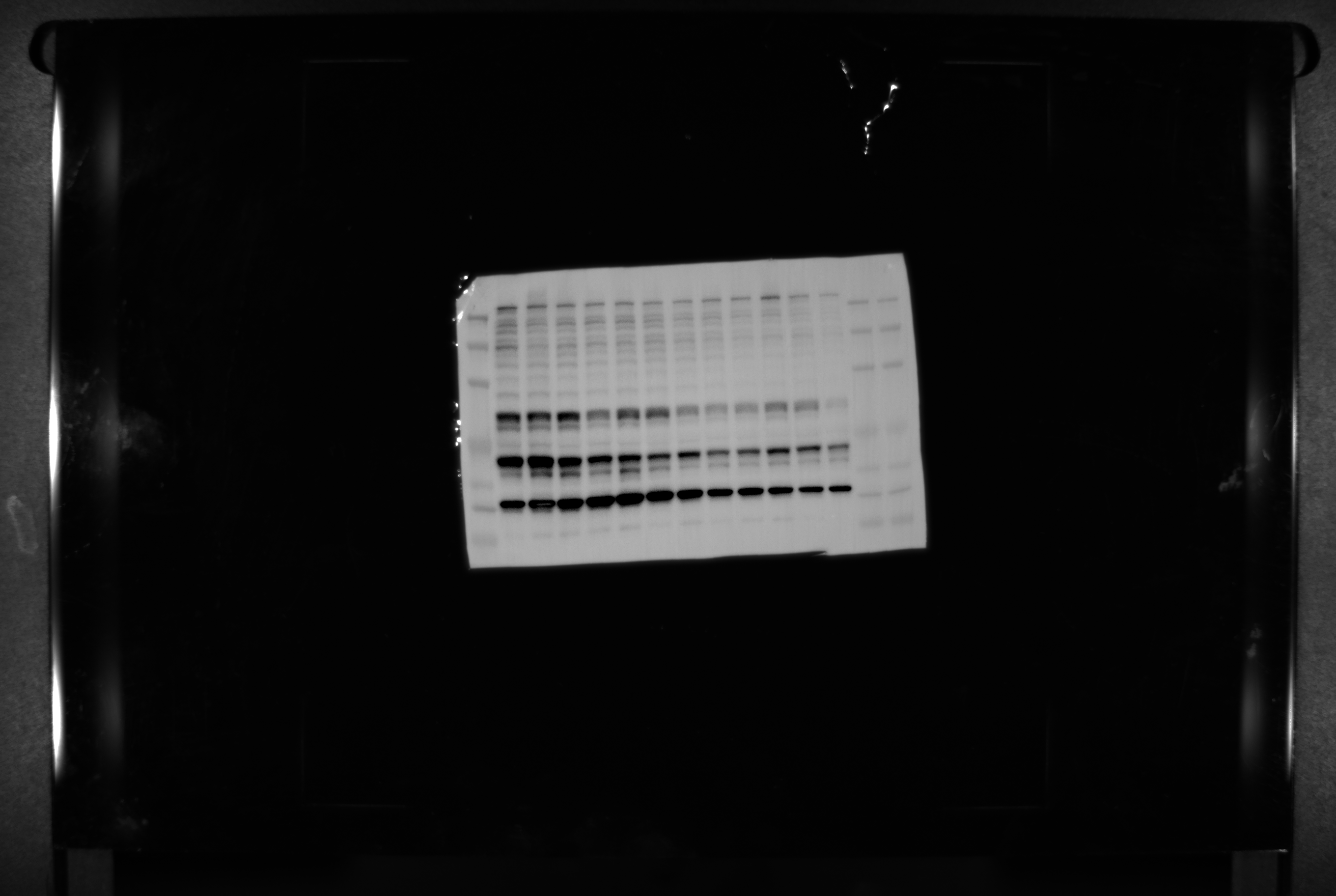

Supplement: Supplementary file 13 [file Image12.tiff]

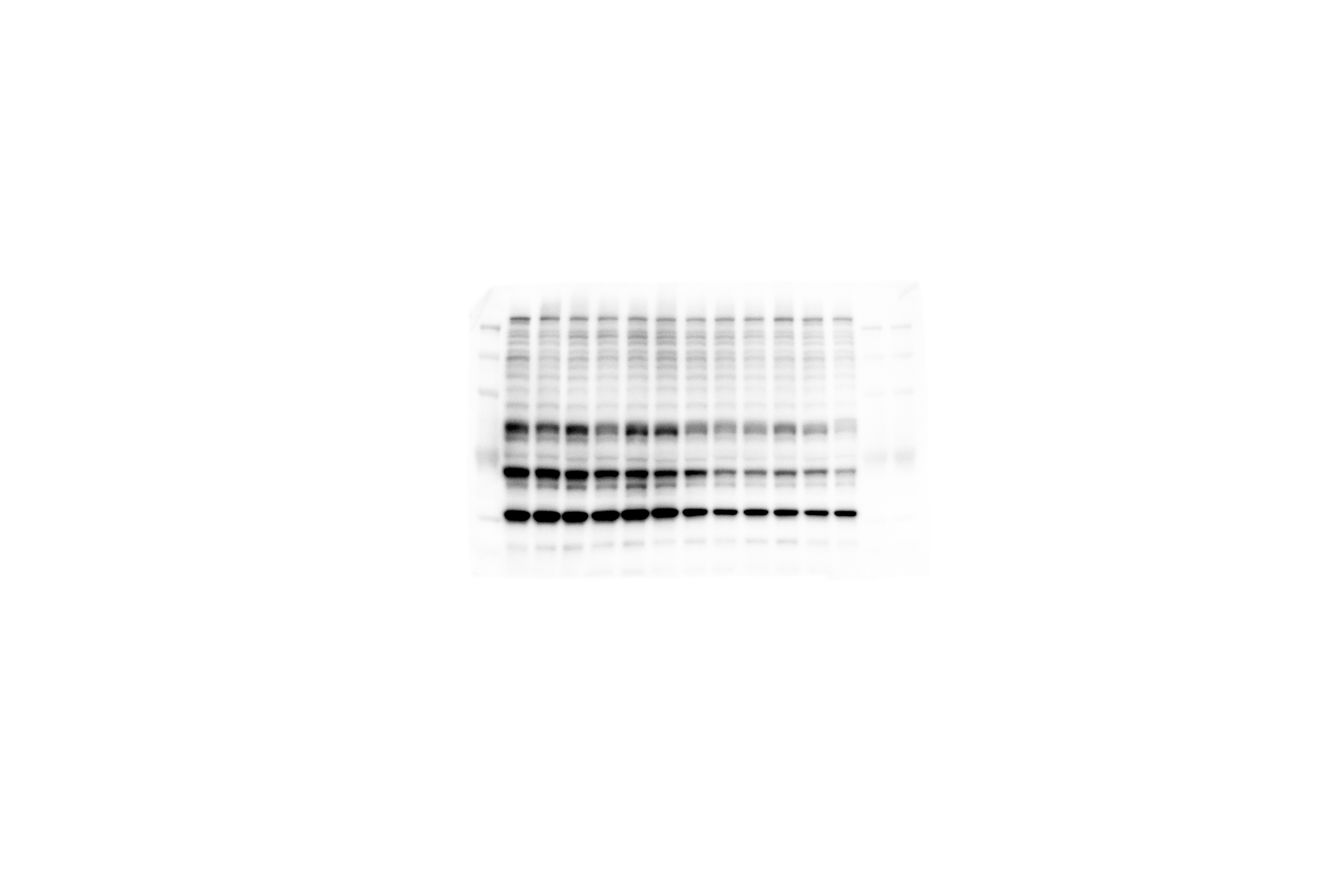

Supplement: Supplementary file 14 [file Image13.tiff]

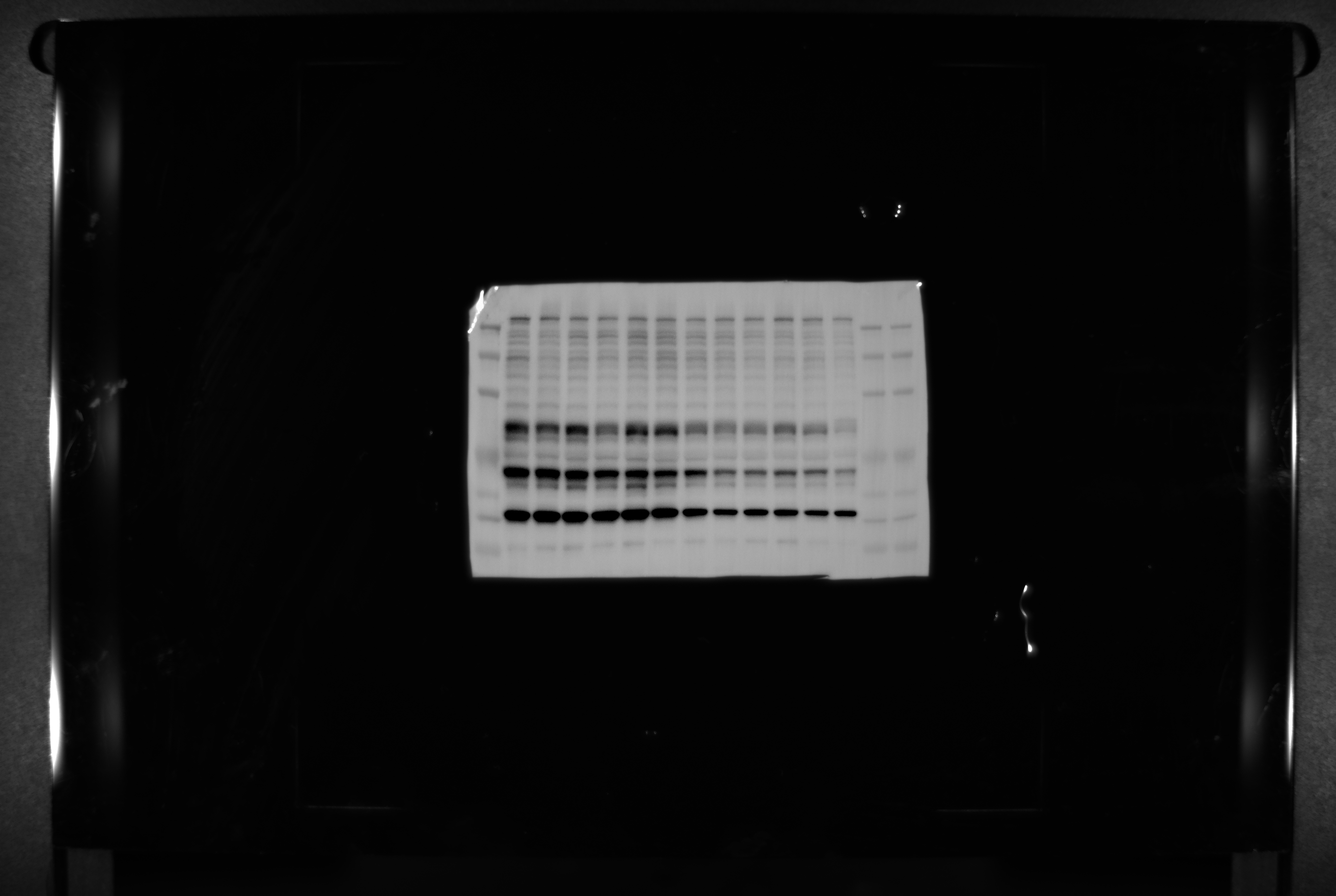

Supplement: Supplementary file 15 [file Image14.tiff]
